# Anti-Inflammatory Triterpenoids from the Stems of *Microtropis Fokienensis*

**DOI:** 10.3390/molecules19044608

**Published:** 2014-04-14

**Authors:** I-Hsiao Chen, Ying-Chi Du, Tsong-Long Hwang, I-Fen Chen, Yu-Hsuan Lan, Hsin-Fu Yen, Fang-Rong Chang, Yang-Chang Wu

**Affiliations:** 1 School of Chinese Medicine for Post Baccalaureate, College of Medicine, I Shou University, Kaohsiung 82445, Taiwan; E-Mail: fantasysp@isu.edu.tw; 2 Department of Botanicals, Medical and Pharmaceutical Industry Technology and Development Center, New Taipei City 248, Taiwan; E-Mail: ycdu0626@gmail.com; 3 Graduate Institute of Natural Products, College of Medicine, Chang Gung University, Taoyuan 333, Taiwan; E-Mail: htl@mail.cgu.edu.tw; 4 Department of Biomedical Engineering, College of Medicine, I Shou University, Kaohsiung 82445, Taiwan; E-Mail: ifen@isu.edu.tw; 5 School of Pharmacy, College of Pharmacy, China Medical University, Taichung 404, Taiwan; E-Mail: lanyh@mail.cmu.edu.tw; 6 National Museum of Natural Science, Taichung 404, Taiwan; E-Mail: hfyen@mail.nmns.edu.tw; 7 Graduate Institute of Natural Products, College of Pharmacy, Kaohsiung Medical University, Kaohsiung 807, Taiwan; 8 Department of Marine Biotechnology and Resources, National Sun Yat-sen University, Kaohsiung 804, Taiwan; 9 Cancer Center, Kaohsiung Medical University Hospital, No. 100 Tz-You First Road, Kaohsiung 807, Taiwan; 10 Chinese Medicine Research and Development Center, China Medical University Hospital, Taichung 404, Taiwan; 11 Center for Molecular Medicine, China Medical University Hospital, Taichung 404, Taiwan

**Keywords:** *Microtropis fokienensis*, triterpenoids, ursane, oleanane, anti-inflammatory

## Abstract

Three new ursane- and four new oleanane- type triterpenoids **1**–**7** were isolated, along with six known compounds **8**–**13**, from the methanolic extract of *Microtropis fokienensis*. All structures were elucidated by mass and NMR spectroscopic methods. The isolates **4**–**10** and known compounds **14**–**17** that were previously isolated from this material were evaluated for anti-inflammatory activity based on effects against superoxide anion generation and elastase release by neutrophils in response to fMLP/CB. 11*α*,30-Dihydroxy-2,3-*seco*-olean-12-en-2,3-dioic anhydride (**7**) was the first triterpene anhydride from the genus of *Microtropis* to have the ring A expanded to a seven-membered ring; it showed significant anti-inflammatory activity against superoxide anion generation and elastase release. Unexpectedly, 30-hydroxy-2,3-*seco*-lup-20(29)-ene-2,3-dioic acid (**17**) showed the best effect against superoxide anion generation and elastase release with IC_50_ values of 0.06 ± 0.01 and 1.03 ± 0.35 µg/mL, respectively. Compound **17** had a dioic acid function, and compound **7** had an anhydride function modification in ring A; both showed promising activity in the target assays.

## 1. Introduction

**Figure 1:**
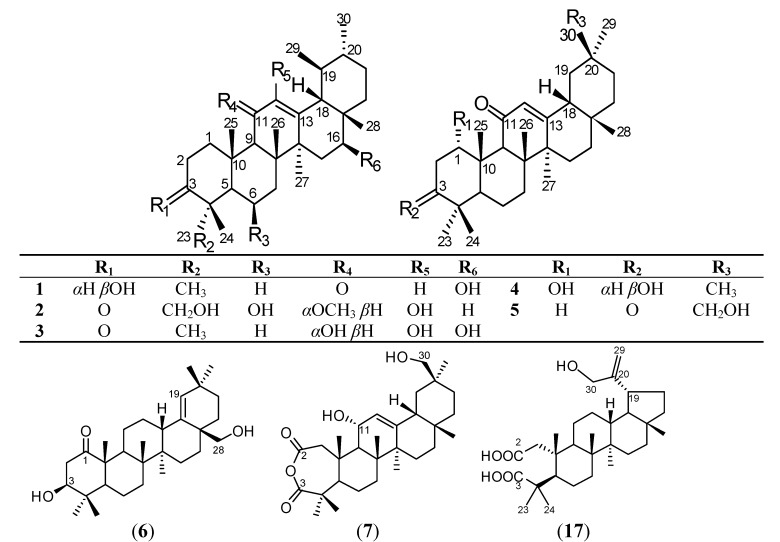
Structures of compounds **1**–**7** and **17**.

Plants belonging to the genus *Microtropis* are members of the family Celastraceae and are evergreen shrubs that are widely distributed in India, Malaysia, Mainland China, Japan, Central America and Mexico [1]. The chemical constituents of the *Microtropis* species in Taiwan include sesquiterpenoids, diterpenoids, and triterpenoids [2,3,4,5]. Pentacyclic triterpenoids are the dominant constituents within the genus *Microtropis* and have a basic framework that is similar to that of oleanane, ursane or lupane. Previously, we reported various cytotoxic triterpenoids that worked against cancer cell lines isolated from *M. fokienensis* and *M. japonica* [4,5,6]. There are no prior reports on the anti-inflammatory effects of metabolites from the genus *Microtropis*. In this study, a bioassay-guided fractionation of a MeOH extract of *M. fokienensis* stems resulted in the isolation of seven new triterpenoids **1**–**7** (Figure 1), and six known compounds **8**–**13**. The isolation and structural elucidation of these triterpenes are reported herein. Because of the limited amounts of samples, compounds **4**–**10** and known compounds, such as 13*β*,28-epoxy-3*β*-hydroxyurs-11-ene (**14**) [7,8], 28,30-dihydroxylup-20(29)-en-3-one (**15**) [9], 30-hydroxybetulin (**16**) [9], and 30-hydroxy-2,3-*seco*-lup-20(29)-ene-2,3-dioic acid (**17**), which were previously isolated from the stems of *M. fokienensis* [4,6], were selected and evaluated for anti-inflammatory activity based on their inhibition of superoxide anion generation and elastase release by human neutrophils in response to fMLP/CB.

## 2. Results and Discussion

The dried and powdered stems of *M. fokienensis* were extracted with methanol. The methanolic extract was concentrated, and the residue was partitioned between ethyl acetate and water to provide an organic extract containing triterpenes and an aqueous extract. The organic extract was further divided into *n*-hexane and aqueous MeOH layers using *n*-hexane and 80% MeOH. The aqueous MeOH layer was separated using column chromatography and purified by RP-HPLC, to obtain seven new triterpenoids: 3*β*,16*β*-dihydroxyurs-12-en-11-one (**1**), 6*β*,12,23-trihydroxy-11*α*-methoxyurs-12-en-3-one (**2**), 11*α*,12,16*β*-trihydroxyurs-12-en-3-one (**3**), 1*α*,3*β*-dihydroxyolean-12-en-11-one (**4**), 30-hydroxyolean-12-en-3,11-dione (**5**), 3*β*,28-dihydroxyolean-18-en-1-one (**6**), and 11*α*,30-dihydroxy-2,3-*seco*-olean-12-en-2,3-dioic anhydride (**7**), as well as six known compounds, which were identified by comparing of their NMR data with those reported in literature as 2*α*,3*β*-dihydroxyurs-12-en-28-oic acid (**8**) [10,11], 3*β*-acetoxyurs-12-en-28-oic acid (**9**) [12], olean-11,13(18)-dien-3*β*,30-diol (**10**) [13], 13*β*,28-epoxy-3*β*,16*β*-dihydroxyurs-11-ene (**11**) [4], 2*α*,3*β*-dihydroxyolean-12-en-28-oic acid (**12**) [14], and olean-12-en-3*β*,30-diol (**13**) [15].

Compound **1** was obtained as a white amorphous solid. Its IR spectrum showed absorption bands for hydroxyl groups (3425 cm^−1^) and an *α*,*β*-unsaturated ketone system (1655 cm^−1^). The molecular formula was determined to be C_30_H_48_O_3_ on the basis of the HRESIMS molecular ion at *m*/*z* 479.3501 ([M+Na]^+^, calcd. for 479.3500), which accounted for seven degrees of unsaturation. The ^1^H-NMR spectrum exhibited an olefinic proton at *δ*_H_ 5.85 (1H, s), two methine protons at *δ*_H_ 3.50 (m) and 4.59 (ddd, *J* = 11.0, 5.2, and 4.4 Hz), five tertiary methyl protons at *δ*_H_ 1.06, 1.09, 1.26, 1.27, and 1.38 (each 3H, s), and two secondary methyl protons at *δ*_H_ 0.80 (3H, d, *J* = 6.4 Hz) and 0.91 (3H, d, *J* = 6.0 Hz) (Table 1). The ^13^C-NMR spectrum also showed an *α*,*β*-unsaturated ketone system (*δ*_C_ 130.7, 163.6, and 199.3), two oxygenated methines at *δ*_C_ 64.7 and 77.9, and eight methyl signals at *δ*_C_ 16.6, 17.0, 17.6, 18.7, 21.2, 21.9, 23.0, and 28.7 (Table 2).

**Table 1:** ^1^H-NMR spectroscopic data for compounds **1**–**7** (in C_5_D_5_N, 400 MHz).

No.	1	2	3 ^a^	4	5	6	7
1	1.20 (m, *α*) 3.14 (dt, 13.6, 3.6, *β*)	1.80 (m, *α*) 2.84 (m, *β*)	2.04 (m, *α*) 3.04 (m, *β*)	5.37 (s)	1.55 (m, *α*) 3.20 (m, *β*)		3.14 (d, 17.6) 3.33 (d, 17.6)
2	1.88 (m, *α*) 2.04 (m, *β*)	2.52 (m, *α*) 2.91 (m, *β*)	2.55 (m, *α*) 2.62 (m, *β*)	2.33 (dt, 13.6, 3.6, *α*) 2.48 (dd, 13.6, 12.0, *β*)	2.46 (m, *α*) 2.69 (m, *β*)	2.74 (dd, 11.6, 4.8, *α*) 3.50 (dd, 12.0, 11.6, *β*)	
3	3.50 (m, *α*)			4.43 (dd, 12.0, 3.6, *α*)		3.75 (dd, 12.0, 4.8, *α*)	
5	0.87 (m)	2.58 (brs)	1.59 (m)	1.71	1.41 (m)	0.98 (dd, 11.2, 2.4)	1.96 (m)
6	1.56 (2H, m)	4.94 (brs, *α*)	1.44 (2H, m)	1.61 (m) 1.72 (m)	1.43 (2H)	1.50 (m) 1.58 (m)	1.58 (m) 1.67 (m)
7	1.71 (m, *α*) 1.40 (m, *β*)	1.98 (m, *α*) 1.86 (m, *β*)	1.66 (m, *α*) 1.46 (m, *β*)	1.74 (m, *α*) 1.35 (m, *β*)	1.30 (m) 1.60 (m)	1.26 (dd, 12.0, 3.2) 1.40 (m)	1.33 (m) 1.73 (m)
9	2.55 (s)	2.36 (d, 10.0)	2.19 (d, 10.0)	3.87 (s)	2.55 (s)	2.27 (dd, 12.0, 2.0)	2.19 (d, 11.2)
11		4.82 (d, 10.0, *β*)	4.58 (d, 9.5, *β*)			1.22 (m) 1.93 (m)	4.91 (dd, 10.8, 2.4)
12	5.85 (s)			5.76 (s)	5.83 (s)	1.43 (m)1.51 (m)	5.49 (d, 2.0)
13						2.40 (m)	
15	1.79 (m, *α*) 2.11 (m, *β*)	1.38 (2H, m)	1.71 (m, *α*) 2.13 (m, *β*)	1.07 1.75	1.09 (m) 1.72 (m)	1.13 (m) 1.89 (m)	0.95 (m) 1.74 (m)
16	4.59 (ddd, 11.0, 5.2, 4.4)	1.43 (2H, m)	4.63 (m, *α*)	1.97 (td, 13.6, 4.4, *α*) 0.83 (m, *β*)	2.10 (td, 13.6, 4.4, *α*) 0.92 (dt, 13.6, 2.0, *β*)	1.32 (m) 2.21 (m)	0.89 (m) 2.04 (td, 13.2, 4.0)
18	1.79 (d, 11.2)	2.90 (m)	3.10 (d, 11.5)	2.11 (dd, 13.6, 4.0)	2.30 (dd, 13.4, 4.4)		2.21 (dd, 10.8, 4.4)
19	1.55 (m)	1.81 (m)	1.68 (m)	0.79 (m) 1.53 (m)	1.52 (m) 1.69 (m)	5.11 (s)	1.60 (m) 1.75 (m)
20	0.91 (m)	2.52 (m)	1.80 (m)				
21	1.46 (m, 2H)	1.38 (2H, m)	1.51 (m) 1.59 (m)	1.06 (m) 1.34 (m)	1.40 (m) 1.69 (m)	1.38 (m) 1.70 (m)	1.32 (m) 1.70 (m)
22	1.13 (d, 3.2, *α*) 2.57 (dt, 13.6, 3.2, *β*)	0.80 (m) 1.96 (m)	1.29 (m) 2.68 (brd, 13.0)	1.22 (m) 1.43 (m)	1.28 (m) 1.56 (m)	1.39 (m) 2.36 (m)	1.32 (m) 1.70 (m)
23	1.26 (s)	3.93 (d, 10.4) 4.47 (d, 10.4)	1.20 (s)	1.38 (s)	1.08 (s)	1.18 (s)	1.40 (s)
24	1.09 (s)	1.70 (s)	1.13 (s)	1.18 (s)	1.18 (s)	1.22 (s)	1.47 (s)
25	1.38 (s)	1.92 (s)	1.27 (s)	1.46 (s)	1.35 (s)	1.29 (s)	1.39 (s)
26	1.27 (s)	1.84 (s)	1.30 (s)	1.22 (s)	1.12 (s)	1.04 (s)	1.01 (s)
27	1.47 (s)	1.31 (s)	1.54 (s)	1.36 (s)	1.37 (s)	0.94 (s)	1.27 (s)
28	1.06 (s)	0.98 (s)	1.26 (s)	0.85 (s)	0.85 (s)	3.81 (d, 10.4) 4.02 (d, 10.4)	0.86 (s)
29	0.80 (d, 6.4)	1.26 (d, 6.4)	1.18 (d, 6.5)	0.81 (s)	1.17 (s)	1.06 (s)	1.16 (s)
30	0.91 (d, 6.0)	0.97 (d, 7.2)	0.95 (d, 6.5)	0.83 (s)	3.74 (d, 10.4) 3.80 (d, 10.4)	1.07 (s)	3.74 (d, 10.4) 3.82 (d, 10.4)

*^a^* Measured in C_5_D_5_N, 500 MHz.

**Table 2:** ^13^C-NMR Spectroscopic Data for Compounds **1**–**7** (in C_5_D_5_N, 100 MHz).

No.	1	2	3 *^a^*	4	5	6	7
1	39.8	40.5	42.5	72.3	40.0	212.9	49.6
2	28.1	36.1	35.1	35.5	34.4	45.4	170.6
3	77.9	216.0	217.2	72.3	215.9	79.0	182.0
4	39.8	54.7	48.3	40.1	47.8	40.1	45.4
5	55.3	49.2	56.1	47.8	55.1	54.9	55.2
6	18.0	67.8	20.5	17.8	19.0	18.1	20.7
7	33.2	41.6	34.4	32.8	32.1	34.4	33.4
8	45.7	42.6	43.5	45.2	45.3	40.7	41.1
9	61.3	46.6	53.8	53.8	61.2	42.8	45.9
10	37.4	37.8	38.5	42.1	37.0	53.5	38.5
11	199.3	77.3	70.2	200.9	198.9	24.4	74.5
12	130.7	145.0	148.4	128.6	128.4	26.7	121.4
13	163.6	116.5	113.2	169.7	170.6	39.6	149.8
14	45.8	41.4	44.2	44.0	43.7	43.7	43.1
15	37.1	27.6	37.8	26.7	26.7	27.8	25.8
16	64.7	28.0	65.9	26.5	26.9	31.3	27.5
17	39.2	33.7	39.5	32.5	32.5	32.5	32.9
18	60.7	47.5	49.9	47.6	47.3	140.7	47.2
19	39.1	41.3	41.9	45.2	40.8	132.6	41.6
20	39.4	40.0	40.7	31.0	36.0	32.5	36.1
21	30.8	31.6	31.8	34.5	29.9	33.4	30.0
22	35.4	42.2	36.8	36.7	36.4	31.1	36.6
23	28.7	66.8	27.5	28.8	21.5	28.6	28.2
24	16.6	20.5	22.1	16.2	26.5	16.6	23.3
25	17.0	17.6	16.9	18.0	15.9	16.1	17.7
26	18.7	20.3	18.9	19.1	18.5	17.0	17.1
27	21.9	24.0	26.0	23.6	23.4	15.1	25.5
28	23.0	28.9	23.6	28.8	28.7	63.6	28.4
29	17.6	17.4	17.8	33.0	28.2	31.4	28.2
30	21.2	21.5	22.0	23.5	65.3	29.7	65.6
OCH_3_		51.4					

*^a^* Measured in C_5_D_5_N, 125 MHz.

**Figure 2:**
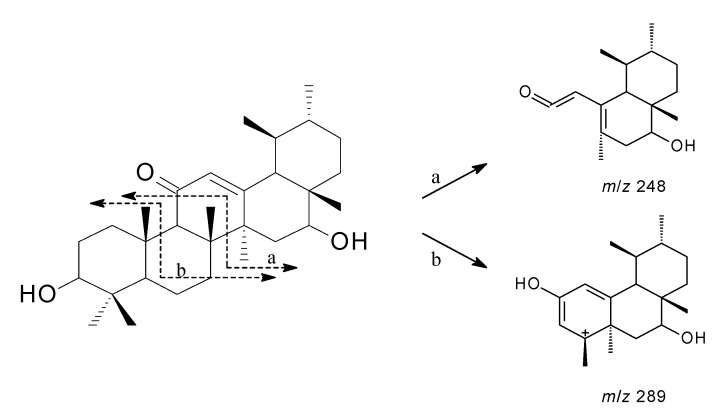
Mass fragmentation patterns of **1**.

**Figure 3:**
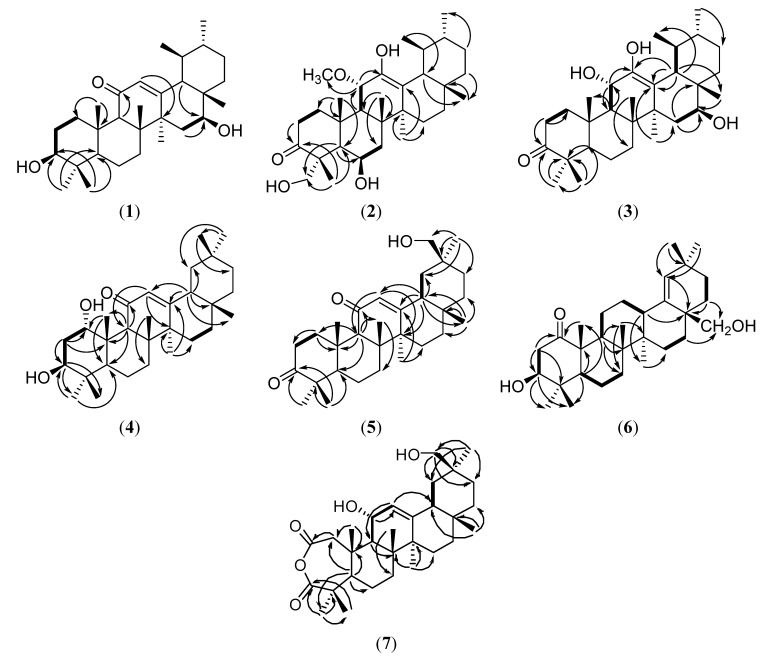
COSY and HMBC correlations of **1**–**7**.

**Figure 4:**
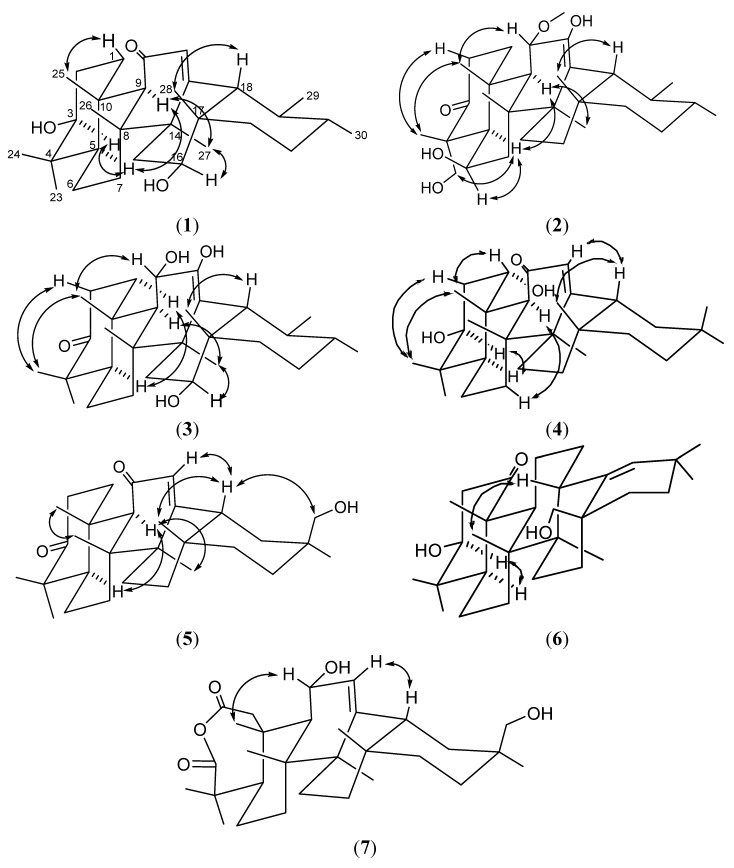
NOESY correlations of **1**–**7**.

Therefore, **1** was suggested to have an urs-12-ene frame and the *α*,*β*-unsaturated ketone system in ring C was similar to 3*β*-hydroxyurs-12-en-11-one, except for the presence of one more oxymethine [16,17,18]. The MS fragmentations at *m*/*z* 248, where each bond was cleaved at the *α*,*β*-unsaturated ketone system in ring C, indicated that the other hydroxyl group was seemed to be located in either ring D or E. (Figure 2) [19]. The hydroxyl group was assigned to C-16, because of the low field shifted signals of *δ*_C_ 60.7 (C-18), and the HMBC correlation of H-18 (*δ*_H_ 1.79) and H-15 (*δ*_H_ 2.11)/C-16 (*δ*_C_ 64.7) (Figure 3). The NOESY experiment showed the NOE effect of H-3 and H-16 with H-5 and H-27 (Figure 4), respectively, indicating that the relative stereochemistry of the two hydroxyl groups at C-3 and C-16 were resolved as the *β* form. Therefore, the structure of **1** was determined and named 3*β*,16*β*-dihydroxyurs-12-en-11-one.

Compound **2** was isolated as a white amorphous powder. HRESIMS indicated a molecular formula of C_31_H_50_O_5_ (*m*/*z* 525.3556 [M+Na]^+^, calcd. for 525.3559). It showed absorption bands in its IR spectrum at 3446, 1694, and 1649 cm^−1^ for hydroxyl, carbonyl, and olefinic functions. The ^1^H-NMR spectrum of **2** exhibited signals due to five tertiary methyls (*δ*_H_ 0.98, 1.31, 1.70, 1.84 and 1.92), two secondary methyls [*δ*_H_ 0.97 (d, *J* = 7.2 Hz) and 1.26 (d, *J* = 6.4 Hz)], a methoxyl (*δ*_H_ 3.35), a hydroxymethylene [*δ*_H_ 3.93 (d, *J* = 10.4 Hz) and 4.47 (d, *J* = 10.4 Hz)], and two oxygenated methines [*δ*_H_ 4.82 (d, *J* = 10.0 Hz) and 4.94 (brs)] (Table 1). The ^13^C-NMR and the DEPT spectra of **2** suggested the presence of a cyclic ketone at *δ*_C_ 216.0, two olefinic carbons at *δ*_C_ 116.5 and 145.0, two oxygenated carbons at *δ*_C_ 67.8 and 77.3, a methylene carbon at *δ*_C_ 66.8, and a methoxy carbon at *δ*_C_ 51.4 (Table 2). Based on the molecular formula of C_31_H_50_O_5_, the degrees of unsaturation of **2** were determined to be seven, including one ketone and double bond. This spectral evidence suggested that **2** was a derivative of the pentacyclic triterpenoid urs-12-en-3-one, with a tetrasubstituted double bond, two oxygenated methines, one hydroxymethylene, and one methoxy moiety [5]. Based on the evidence of the HMBC spectrum (Figure 3), a secondary hydroxyl, a methoxy, and a tertiary hydroxyl groups were deemed to be attached at C-6, C-11, and C-12. The chemical shifts of C-4 (*δ*_C_ 54.7) and CH_3_-24 (*δ*_C_ 20.5) led to the assignment of the CH_2_OH unit at the C-23*α* position [20]. The NOESY (Figure 4) correlations illustrated the stereochemistry of **2**. Therefore, the structure of the new compound **2** was determined to be 6*β*,12,23-trihydroxy-11*α*-methoxyurs-12-en-3-one.

Compound **3**, isolated as white amorphous powder, showed IR absorption bands for hydroxyl (3418 cm^−1^) and carbonyl (1694 cm^−1^) groups. The molecular formula was determined as C_30_H_48_O_4_ based on HRESIMS (495.3450 [M+Na]^+^, calcd. for 495.3452), corresponding to seven degrees of unsaturation. The ^1^H-NMR spectrum of **3** exhibited signals that were due to six tertiary methyls [*δ*_Η_ 1.13, 1.20, 1.26, 1.27, 1.30, and 1.54], two secondary methyls [*δ*_Η_ 0.95 (d, *J* = 6.5 Hz) and 1.18 (d, *J* = 6.5 Hz)], and two oxygenated methines [*δ*_Η_ 4.58 (d, *J* = 9.5 Hz) and 4.63 (m)] (Table 1). The ^13^C-NMR spectrum indicated that **3** was constituted by 30 carbons including a cyclic ketone [*δ*_C_ 217.2], two olefinic carbons [*δ*_C_ 113.2 and 148.4], and two oxygenated carbons [*δ*_C_ 65.9 and 70.2] (Table 2). This spectral evidence suggested that **3** was an urs-12-en-3-one derivative with a tetrasubstituted double bond, and two oxygen-bearing functional groups [5]. The partial structure of **3** was solved by the HMBC correlations of the eight methyl groups. Detailed analysis of other correlations in the HMBC spectrum determined the locations of the followings substituents: three hydroxyl groups were located at C-11, C-12, and C-16, and a double bond between C-12 and C-13 (Figure 3). The stereochemistry of **3** was further established from the NOESY spectrum (Figure 4), and the structure 11*α*,12,16*β*-trihydroxyurs-12-en-3-one was assigned.

Compound **4** was assigned a molecular formula of C_30_H_48_O_3_ and seven degrees of unsaturation, as deduced from the HRESIMS (*m*/*z* 479.3501 [M+Na]^+^, calcd. for 479.3503) and ^13^C-NMR spectra. The IR spectrum showed a hydroxyl group (3421 cm^−1^) and an *α*,*β*-unsaturated ketone system (1652 cm^−1^). The ^13^C-NMR and DEPT spectra displayed 30 carbon signals, with signals for two carbons bearing oxygen observed at *δ*_C_ 72.3 (2 × C) and signals for an *α*,*β*-unsaturated ketone system observed at *δ*_C_ 128.6, 169.7, and 200.9. A comparison of the ^1^H and ^13^C-NMR spectra data of **4** with those of **1** (Table 1 and Table 2) revealed that **4** differed from **1** in the presence of eight tertiary methyls and had an oleanane skeleton [21]. The substituted functions and planar structure of **4** were established in extensive interpretation of its 2D NMR spectra (Figure 3). Based on the NOESY spectrum (Figure 4), the configurations of the H-1 and H-3 were assigned to be *β* and *α* orientation. Therefore, the structure of **4** was determined to be 1*α*,3*β*-dihydroxyolean-12-en-11-one.

The IR spectrum of compound **5** showed a hydroxyl group (3441 cm^−1^), a carbonyl group (1697 cm^−1^), and an *α*,*β*-unsaturated ketone system (1648 cm^−1^). The molecular formula C_30_H_46_O_3_ was established by HRESIMS (*m*/*z* 477.3344 [M+Na]^+^, calcd. for 477.3341), implying eight degrees of unsaturation. The characteristic ^1^H and ^13^C-NMR data of **5** (Table 1 and Table 2) indicated the presence of a carbonyl and a hydroxymethylene on the 12-oleanen-11-one chemical frame instead of two hydroxymethines in **4** [19]. The key HMBC were shown on Figure 3 in order to determine the locations of the substituents and the planar structure. Based on this evidence and the NOESY (Figure 4), a primary alcohol group was deemed to be located at C-30. Therefore, the structure of compound **5** was determined to be 30-hydroxyolean-12-en-3,11-dione.

The molecular formula of **6** was assigned as C_30_H_48_O_3_ (*m*/*z* 479.3501 [M+Na]^+^, calcd. for 479.3504) by HRESIMS. The IR spectrum indicated the occurrence of hydroxyl (3376 cm^−1^), carbonyl (1698 cm^−1^), and olefinic (1632 cm^−1^) moieties. The ^1^H and ^13^C-NMR spectra of **6** exhibited signals (Table 1 and Table 2) for seven tertiary methyls, a hydroxymethylene [*δ*_H_ 3.81 and 4.02 (1H each, *J* = 10.4 Hz); *δ*_C_ 63.6], an oxygenated-methine [*δ*_H_ 3.75 (1H, m); *δ*_C_ 79.0], and a trisubstituted double bond [*δ*_H_ 5.11 (1H, brs); *δ*_C_ 132.6 and 140.7]. The ^1^H and ^13^C-NMR spectra and 2D NMR experiments revealed features of the Δ^18^ oleanane-type triterpene with one carbonyl and two hydroxyl groups [22,23,24,25]. The characteristic signals including an upfield shift of C-29 (Δ 2 ppm) and a downfield shift of C-30 (Δ 7 ppm) in the ^13^C-NMR spectrum confirmed that **6** is an Δ^18^ oleanane-type triterpene [26]. The COSY, HMQC, and HMBC determined the locations of the substituents and the planar structure, and a carbonyl, an oxygenated-methine, and a hydroxymethylene were assigned at C-1, C-3, and C-28 (Figure 3). The coupling constants of the oxygenated methine at *δ*_H_ 3.75 (*J* = 12.0, 4.8 Hz) indicated a 3*β*-hydroxyl group [27]. Accordingly, compound **6** was determined to be 3*β*,28-dihydroxyolean-18-en-1-one.

Compound **7** was isolated as a white amorphous powder. Its molecular formula, C_30_H_46_O_5_, was determined by HRESIMS (*m*/*z* 509.3243 [M+Na]^+^, calcd. for 509.3246), corresponding to eight degrees of unsaturation. The IR spectrum showed absorption bands for hydroxyl (3426 cm^−1^) and carbonyl (1712 cm^−1^, broad) functional groups. The ^1^H-NMR spectrum revealed signals due to an olefinic proton [*δ*_H_ 5.49 (d, *J* = 2.0 Hz)], seven tertiary methyls (*δ*_H_ 0.86, 1.01, 1.16, 1.27, 1.39, 1.40, and 1.47), a hydroxymethylene [*δ*_H_ 3.74 (d, *J* = 10.4 Hz), 3.82 (d, *J* = 10.4 Hz)], and an oxygenated methines [*δ*_H_ 4.91 (dd, *J* = 10.8, 2.4 Hz)] (Table 1). The ^13^C-NMR spectrum indicated the presence of 30 carbon atoms, including one trisubstituted double bond (*δ*_C_ 121.4 and 149.8), seven methyls, nine methylenes (one oxygenated), four methines (one oxygenated), and eight quaternary carbons (two carbonyl carbons) (Table 2). Previous studies [28,29] showed two carbonyls groups at *δ*_C_ 170.6 and 182.0 together with the absence of absorption bands for carboxylic groups in the IR spectrum, which indicated the presence of an anhydride moiety in the molecule. Eight degrees of unsaturation were implied by the molecular formula. As well as two carbonyls and one olefinic group, compound **7** possesses a pentacyclic system. This data indicated that this compound belongs to the olean-12-ene family of triterpenoids, with an embedded anhydride function and two additional hydroxyl groups [28,29]. In the HMBC spectrum of **7**, the correlations for H-11 (*δ*_H_ 4.91)/C-12 (*δ*_C_ 121.4), and for H-12 (*δ*_H_ 5.49)/C-9 (*δ*_C_ 45.9) and C-18 (*δ*_C_ 47.2) indicated that a hydroxyl group was attached at C-11. The HMBC correlations of both H-29 and H-30 with C-19 and C-20 also indicated that the hydroxymethylene group was attached at C-20. On the basis of the previous study [30], the chemical shift of the C-29 (equatorial) hydroxymethylene group resonated at around 75 ppm and the value of the C-30 (axial) methyl group was found at around 20 ppm in the ^13^C-NMR spectrum. In contrast, the chemical shift of the C-30 hydroxymethylene group appeared at around 67 ppm, while the C-29 methyl group appeared at around 28 ppm. Therefore, the remaining hydroxyl group in **7** was placed at C-30. Detailed analysis of the correlations of the HMBC spectrum gave the location of the anhydride at C-2 and C-3 (Figure 3). The orientation of a hydroxyl group at C-11 was assumed to be *α* on the basis of the NOESY correlations between H-11 and H-25. In according with all these data, the structure of **7** was established as 11*α*,30-dihydroxy-2,3-*seco*-olean-12-en-2,3-dioic anhydride.

Although a triterpene anhydride has been reported from species of the Celastraceae family, 11*α*,30-dihydroxy-2,3-*seco*-olean-12-en-2,3-dioic anhydride (**7**) represents the first example of a triterpene anhydride with the ring A expanding to a seven-membered ring from the genus of *Microtropis*.

As mentioned in the section of introduction, several cytotoxic triterpenoids have been reported to inhibit various cancer cell lines isolated from *M. fokienensis* and *M. japonica* [4,5,6], but there are no prior reports of the anti-inflammatory effects of metabolites from the genus *Microtropis*. A target assay based on effects against superoxide anion generation and elastase release by human neutrophils in response to fMLP/CB was carried out. Due to the limited amounts of samples, **4**–**10** and known compounds, such as 13*β*,28-epoxy-3*β*-hydroxyurs-11-ene (**14**), 28,30-dihydroxylup-20(29)-en-3-one (**15**), 30-hydroxybetulin (**16**), and 30-hydroxy-2,3-*seco*-lup-20(29)-ene-2,3-dioic acid (**17**), which were isolated previously from the stems of *M. fokienensis* [6], were selected and evaluated (Table 3). Compounds **7** and **17** showed significant anti-inflammatory activity against superoxide anion generation and elastase release, with IC_50_ values of 2.10 ± 0.13/2.93 ± 0.27 and 0.06 ± 0.01/1.03 ± 0.35 µg/mL, respectively. Compounds **5** and **6** selectively inhibited elastase release, with an IC_50_ values of 1.53 ± 0.09 and 3.23 ± 0.24 µg/mL, respectively. Compounds **8** and **15** also exhibited significant anti-inflammatory activity against superoxide anion generation, with IC_50_ values of 0.93 ± 0.20 and 2.66 ± 0.78 µg/mL, respectively.

**Table 3:** Inhibitory effects of compounds **4**–**10** and **14**–**17** from *M. fokienensis* on superoxide anion generation and elastase release by human neutrophils in response to fMLP/CB.

Compounds	Superoxide anion	Elastase
	IC_50_ (μg/mL) ^a^ or (Inh %)	IC_50_ (µg/mL) ^a^ or (Inh %)
**4**	125.9 ± 6.72 ^b^ ***	44.62 ± 2.82 ^c^ ***
**5**	19.65 ± 4.23 ^b^ **	1.53 ± 0.09
**6**	(4.35 ± 6.05)	3.23 ± 0.24
**7**	2.10 ± 0.13	2.93 ± 0.27
**8**	0.93 ± 0.20	4.39 ± 1.13
**9**	16.71 ± 3.39 ^b^ **	5.65 ± 0.26
**10**	30.27 ± 5.04 ^b^ ***	5.33 ± 0.73
**14**	(−2.09 ± 4.02)	(14.50 ± 7.26)
**15**	2.66 ± 0.78	3.23 ± 0.88
**16**	3.12 ± 0.60	4.53 ± 0.39
**17**	0.06 ± 0.01	1.03 ± 0.35
DPI ^d^	0.43 ± 0.12	-
PMSF ^d^	-	17.58 ± 5.79

Percentage of inhibition (Inh %) at 10 µg/mL concentration. Results are presented as mean ± S.E.M. (*n* = 3–5). ** *p* < 0.01, *** *p* < 0.001 compared with the control value; ^a^ Concentration necessary for 50% inhibition (IC_50_); ^b^ **4**, **5**, **9**, and **10** induced superoxide anion generation by human neutrophils in absence of fMLP/CB; ^c^ **4** induced elastase release by human neutrophils in present of CB; ^d^ Diphenyleneiodonium (DPI, a NADPH oxidase inhibitor) and phenylmethylsulfonyl fluoride (PMSF, a serine protease inhibitior) were used as the positive controls in the generation of superoxide anion and release of elastase, respectively.

## 3. Experimental

### 3.1. General

The IR spectra were obtained using a Mattson Genesis II spectrometer. ^1^H and ^13^C-NMR spectra were recorded using Varian VNMR-600, Varian INOVA 500, Varian Unity Plus 400, or Varian Gemini 200 NMR spectrometers. Chemical shifts (*δ*) are reported in parts per million, and coupling constants (*J*) are expressed in Hertz. The LREIMS and LRESIMS were measured using a VG Biotech Quattro 5022 mass spectrometer. The HRESIMS were measured using a Bruker Daltonics APEX Π mass spectrometer. Silica gel 60 (Merck, 230–400 mesh) and Sephadex LH-20 were used for column chromatography and TLC analysis was performed on silica gel GF_254_ pre-coated plates and detection used 50% H_2_SO_4_ followed by heating on a hot plate. HPLC was performed using a Hitachi L-7100 pump and a D-7000 interface equipped with a Bischoff RI detector using ODS (Hypersil^®^, 250 × 4 mm; Hypersil^®^, 250 × 10 mm) columns.

### 3.2. Plant Material

The dried stems of *Microtropis fokienensis* were collected from Taichung County, Taiwan, in June 2004, and identified by a botanist, Dr. Hsin-Fu Yen. A voucher specimen (Microtropis-01) was deposited at the Graduate Institute of Natural Products, Kaohsiung Medical University, Kaohsiung, Taiwan.

### 3.3. Extraction and Isolation

The dried stems (2.85 kg) of *M*. *fokienensis* were extracted four times with MeOH (10 L) overnight at room temperature, followed by removal of the solvent under reduced pressure, to yield a dried MeOH extract (130 g). The MeOH extract was dissolved in H_2_O and extracted using EtOAc. Using *n*-hexane and 80% MeOH, the EtOAc-soluble fraction was divided into *n*-hexane and aqueous MeOH layers. The aqueous MeOH layer (38.0 g) was chromatographed on silica gel using mixtures of *n*-hexane–EtOAc of increasing polarity as eluants to afford eighteen fractions. Fr. 10 (2.5 g) was chromatographed on Sephadex LH-20, using CHCl_3_–MeOH (1:1) to give six subfractions. Fr. 10-2-8 was purified using an ODS HPLC column (250 × 10 mm, Hypersil^®^, MeOH–H_2_O, 97:3) to give **5** (14.1 mg, t_R_ 13.4 min, flow rate 2 mL/min). Fr. 10-3 (132.88 mg) was further separated using an ODS HPLC column (250 × 10 mm, Hypersil^®^, MeOH–H_2_O, 78:22) to afford **1** (1.3 mg, t_R_ 24.1 min, flow rate 2 mL/min), **2** (1.9 mg, t_R_ 34.6 min, flow rate 2 mL/min), **3** (1.9 mg, t_R_ 15.4 min, flow rate 2 mL/min), and **6** (2.4 mg, t_R_ 51.1 min, flow rate 2 mL/min). Fr. 12 (5.5 g) was chromatographed on Sephadex LH-20, by elution with CHCl_3_–MeOH (1:1) to give six subfractions. Fr. 12-3 (4.2 g) was subjected to column chromatography over silica gel (CHCl_3_#x2013;MeOH of increasing polarity) to give twelve subfractions. Fr. 12-3-7 (279 mg) was subjected to passage over an ODS HPLC column (250 × 10 mm, Hypersil, MeOH–H_2_O, 87:13) to give **4** (12.1 mg, t_R_ 46.4 min, flow rate 2 mL/min). Fr. 12-3-8-2 (41.67 mg) was further separated using an ODS HPLC column (250 × 10 mm, Hypersil^®^, MeOH–H_2_O, 90:10) to afford **7** (7.3 mg, t_R_ 15.2 min, flow rate 2 mL/min).

*3β,16β-Dihydroxyurs-12-en-11-one* (**1**) was obtained as white amorphous solid; IR (neat) ν_max_ 3425, 2928, 2869, 1726, 1655, 1456, 1385, 1247, 1208 cm^−1^; ^1^H-NMR (C_5_D_5_N, 400 MHz) and ^13^C-NMR (C_5_D_5_N, 100 MHz) see Table 1 and Table 2; EIMS *m*/*z* [M]^+^ 456 (15), 290 (68), 248 (100); HRESIMS *m*/*z* 479.3501 [M+Na]^+^ (calcd. for C_30_H_48_O_3_Na, 479.3500).

*6β,12,23-Trihydroxy-11α-methoxyurs-12-en-3-one* (**2**) was obtained as white amorphous powder; IR (neat) ν_max_ 3446, 2922, 2858, 1694, 1649, 1456, 1376, 1253 cm^−1^; ^1^H-NMR (C_5_D_5_N, 400 MHz) and ^13^C-NMR (C_5_D_5_N, 100 MHz) see Table 1 and Table 2; ESIMS *m*/*z* 525 [M+Na]^+^; HRESIMS *m*/*z* 525.3556 [M+Na]^+^ (calcd. for C_31_H_50_O_5_Na, 525.3559).

*11α,12,16β-Trihydroxyurs-12-en-3-one* (**3**) was obtained as white amorphous powder; IR (neat) ν_max_ 3418, 2923, 2850, 1694, 1455, 1380, 1252 cm^−1^; ^1^H-NMR (C_5_D_5_N, 500 MHz) and ^13^C-NMR (C_5_D_5_N, 125 MHz) see Table 1 and Table 2; ESIMS *m*/*z* 495 [M+Na]^+^; HRESIMS *m*/*z* 495.3450 [M+Na]^+^ (calcd. for C_30_H_48_O_4_Na, 495.3452).

*1α,3β-Dihydroxyolean-12-en-11-one* (**4**) was obtained as white amorphous powder; IR (neat) ν_max_ 3421, 2947, 2856, 1697, 1652, 1458, 1385, 1238 cm^−1^; ^1^H-NMR (C_5_D_5_N, 400 MHz) and ^13^C-NMR (C_5_D_5_N, 100 MHz) see Table 1 and Table 2; ESIMS *m*/*z* 479 [M+Na]^+^; HRESIMS *m*/*z* 479.3501 [M+Na]^+^ (calcd. for C_30_H_48_O_3_Na, 479.3503).

*30-Hydroxyolean-12-en-3,11-dione* (**5**) was obtained as white amorphous powder; IR (neat) ν_max_ 3441, 2923, 2856, 1697, 1648, 1455, 1384, 1202 cm^−1^; ^1^H-NMR (C_5_D_5_N, 400 MHz) and ^13^C-NMR (C_5_D_5_N, 100 MHz) see Table 1 and Table 2; EIMS *m*/*z* [M]^+^ 454 (15), 341 (25), 289 (33), 248 (65); HRESIMS *m*/*z* 477.3344 [M+Na]^+^ (calcd. for C_30_H_46_O_3_Na, 477.3341).

*3β,28-Dihydroxyolean-18-en-1-one* (**6**) was obtained as white amorphous powder; IR (neat) ν_max_ 3376, 2951, 2863, 1698, 1632, 1464, 1385, 1248 cm^−1^; ^1^H-NMR (C_5_D_5_N, 400 MHz) and ^13^C-NMR (C_5_D_5_N, 100 MHz) see Table 1 and Table 2; ESIMS *m*/*z* 479 [M+Na]^+^; HRESIMS *m*/*z* 479.3501 [M+Na]^+^ (calcd. for C_30_H_48_O_3_Na, 479.3504).

*11α,30-Dihydroxy-2,3-seco-olean-12-en-2,3-dioic anhydride* (**7**) was obtained as white amorphous powder; IR (neat) ν_max_ 3426, 2923, 1712, 1454, 1394, 1373, 1263, 1235 cm^−1^; ^1^H-NMR (C_5_D_5_N, 400 MHz) and ^13^C-NMR (C_5_D_5_N, 100 MHz) see Table 1 and Table 2; EIMS *m*/*z* [M]^+^ 486 (18), 341 (19), 267 (26); HRESIMS *m*/*z* 509.3243 [M+Na]^+^ (calcd. for C_30_H_46_O_5_Na, 509.3246).

### 3.4. The Preparation of Human Neutrophils

Blood was taken from healthy human donors (20–35 years old) by venipuncture using a protocol approved by the Institutional Review Board at Chang Gung Memorial Hospital. Neutrophils were isolated using a standard method as previously described [31,32].

### 3.5. The Measurement of Superoxide Generation

SOD inhibition was measured by the reduction of ferricytochrome *c* [31,32]. Neutrophils in 0.5 mg/mL ferricytochrome *c* and 1 mM Ca^2^^+ ^were equilibrated at 37 °C for 2 min and then incubated with drugs for 5 min. The cells were activated using formyl-methionyl-leucyl-phenylalanine (fMLP, 100 nM)/cytochalasin B (CB, 1 µg/mL) for 10 min. The absorbance was continuously monitored at 550 nm using a double-beam, six-cell positioned spectrophotometer with constant stirring (Hitachi U-3010, Tokyo, Japan). Calculations were based on the differences in absorbance with and without SOD (100 µ/mL) divided by the extinction coefficient for the reduction of ferricytochrome c (ε = 21.1/mM/10 mm).

### 3.6. The Measurement of Elastase Release

The degranulation of azurophilic granules was determined by elastase release as described previously [32]. Neutrophils (6 × 10^5^/mL) were equilibrated in MeO-Suc-Ala-Ala-Pro-Val-*p*-nitroanilide (100 µM), an elastase substrate, at 37 °C for 2 min and then incubated with drugs for 5 min. The cells were activated using fMLP (100 nM) in the presence of CB (0.5 µg/mL), and changes in the absorbance at 405 nm were continuously measured to monitor elastase release. The results are expressed as a percentage of the initial rate of elastase release in the fMLP/CB-activated, drug-free control system.

## 4. Conclusions

In this study, a bioassay-guided separation of a MeOH extract of *M. fokienensis* stems resulted in the isolation of seven new triterpenoids **1**–**7** and six known compounds **8**–**13**. Their isolation, purification, and structural determination are reported. Compounds **4**–**10** and **14**–**17** were tested in an anti-inflammatory assay for effects against superoxide anion generation and elastase release by human neutrophils in response to fMLP/CB. Compounds **7** and **17** showed significant anti-inflammatory activity against superoxide anion generation and elastase release. Compounds **5** and **6** exhibited a selective inhibitory effect on elastase release. Compounds **8** and **15** exhibited good anti-inflammatory activity against superoxide anion generation. Interestingly, compound **17** had a dioic acid function and compound **7** had an anhydride function modification in ring A. Both perform well in the target assays. We propose that the modified dicarbonyl A-ring systems of triterpenoids may play a pivotal role in exhibiting anti-inflammatory biological functions on human neutrophils in response to fMLP/CB. This is also the first report of the anti-inflammatory effects of a plant from this genus, and these compounds may lead to pharmaceutical advances in the near future.

## Supplementary Materials

Supplementary materials can be accessed at: http://www.mdpi.com/1420-3049/19/4/4608/s1.
