# Supplementary material for: Anti-Inflammatory Triterpenoids from the Stems of Microtropis Fokienensis
Source: Molecules. 2014 Apr 14;19(4):4608–23. doi: 10.3390/molecules19044608 (PMC6271155; doi:10.3390/molecules19044608)

Supporting Information

Figure	Contents	Pages
**S1.**	Structures of known compounds (**8-16**)	S2
**S2.**	The ^{1}H-NMR spectrum (C_5D_5N, 400 MHz) of 3*β*,16*β*-dihydroxyurs-12-en-11-one (**1**)	S3
**S3.**	The ^{13}C-NMR spectrum (C_5D_5N, 100 MHz) of 3*β*,16*β*-dihydroxyurs-12-en-11-one (**1**)	S3
**S4.**	The COSY spectrum of 3*β*,16*β*-dihydroxyurs-12-en-11-one (**1**)	S4
**S5.**	The HMBC spectrum of 3*β*,16*β*-dihydroxyurs-12-en-11-one (**1**)	S4
**S6.**	The ^{1}H-NMR spectrum (C_5D_5N, 400 MHz) of 6*β*,12,23-trihydroxy-11*α*-methoxyurs-12-en-3-one (**2**)	S5
**S7.**	The ^{13}C-NMR spectrum (C_5D_5N, 100 MHz) of 6*β*,12,23-trihydroxy-11*α*-methoxyurs-12-en-3-one (**2**)	S5
**S8.**	The COSY spectrum of 6*β*,12,23-trihydroxy-11*α*-methoxyurs-12-en-3-one (**2**)	S6
**S9.**	The HMBC spectrum of 6*β*,12,23-trihydroxy-11*α*-methoxyurs-12-en-3-one (**2**)	S6
**S10.**	The ^{1}H-NMR spectrum (C_5D_5N, 500 MHz) of 11*α*,12,16*β*-trihydroxyurs-12-en-3-one (**3**)	S7
**S11.**	The ^{13}C-NMR spectrum (C_5D_5N, 125 MHz) of 11*α*,12,16*β*-trihydroxyurs-12-en-3-one (**3**)	S7
**S12.**	The COSY spectrum of 11*α*,12,16*β*-trihydroxyurs-12-en-3-one (**3**)	S8
**S13.**	The HMBC spectrum of 11*α*,12,16*β*-trihydroxyurs-12-en-3-one (**3**)	S8
**S14.**	The ^{1}H-NMR spectrum (C_5D_5N, 400 MHz) of 1*α*,3*β*-dihydroxyolean-12-en-11-one (**4**)	S9
**S15.**	The ^{13}C-NMR spectrum (C_5D_5N, 100 MHz) of 1*α*,3*β*-dihydroxyolean-12-en-11-one (**4**)	S9
**S16.**	The COSY spectrum of 1*α*,3*β*-dihydroxyolean-12-en-11-one (**4**)	S10
**S17.**	The HMBC spectrum of 1*α*,3*β*-dihydroxyolean-12-en-11-one (**4**)	S10
**S18.**	The ^{1}H-NMR spectrum (C_5D_5N, 400 MHz) of 30-hydroxyolean-12-en-3,11-dione (**5**)	S11
**S19.**	The ^{13}C-NMR spectrum (C_5D_5N, 100 MHz) of 30-hydroxyolean-12-en-3,11-dione (**5**)	S11
**S20.**	The COSY spectrum of 30-hydroxyolean-12-en-3,11-dione (**5**)	S12
**S21.**	The HMBC spectrum of 30-hydroxyolean-12-en-3,11-dione (**5**)	S12
**S22.**	The ^{1}H-NMR spectrum (C_5D_5N, 400 MHz) of 3*β*,28-dihydroxyolean-18-en-1-one (**6**)	S13
**S23.**	The ^{13}C-NMR spectrum (C_5D_5N, 100 MHz) of 3*β*,28-dihydroxyolean-18-en-1-one (**6**)	S13
**S24.**	The COSY spectrum of 3*β*,28-dihydroxyolean-18-en-1-one (**6**)	S14
**S25.**	The HMBC spectrum of 3*β*,28-dihydroxyolean-18-en-1-one (**6**)	S14
**S26.**	The ^{1}H-NMR spectrum (C_5D_5N, 400 MHz) of 11*α*,30-dihydroxy-2,3-*seco*-olean-12-en-2,3-dioic anhydride (**7**)	S15
**S27.**	The ^{13}C-NMR spectrum (C_5D_5N, 100 MHz) of 11*α*,30-dihydroxy-2,3-*seco*-olean-12-en-2,3-dioic anhydride (**7**)	S15
**S28.**	The COSY spectrum of 11*α*,30-dihydroxy-2,3-*seco*-olean-12-en-2,3-dioic anhydride (**7**)	S16
**S29.**	The HMBC spectrum of 11*α*,30-dihydroxy-2,3-*seco*-olean-12-en-2,3-dioic anhydride (**7**)	S16

Figure S1. Structures of known compounds (**8**–**16**).

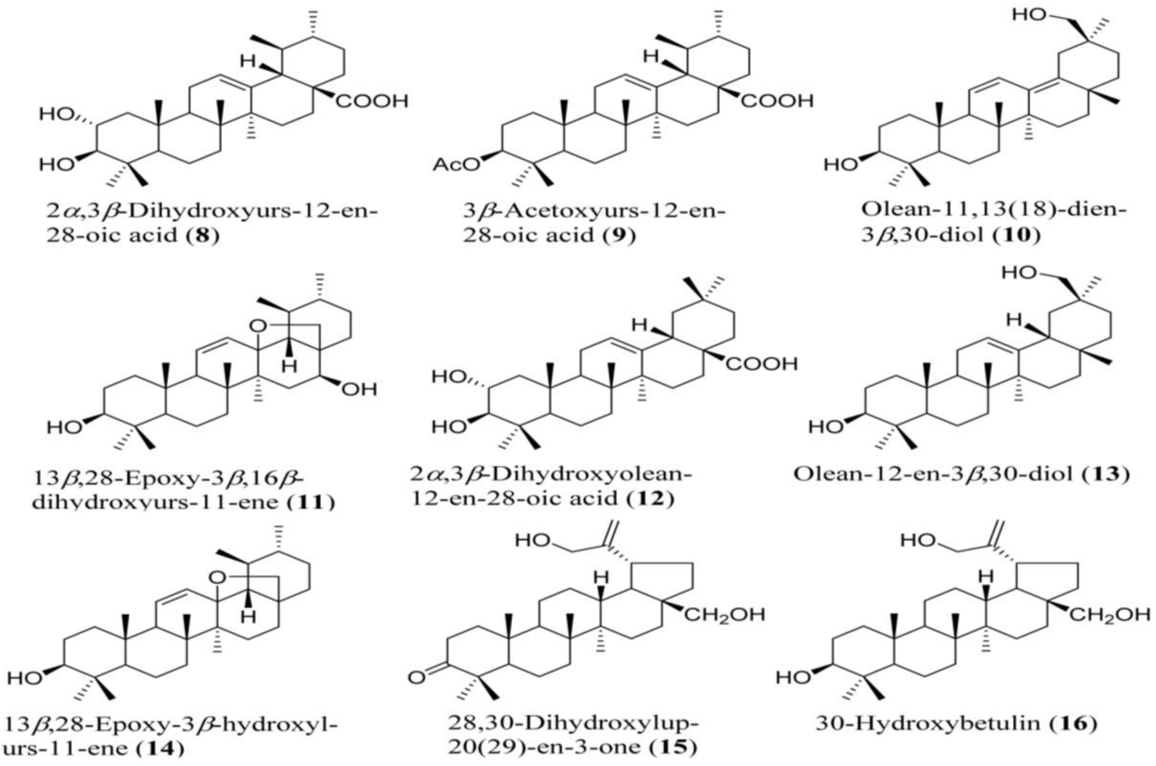

Figure S2. The [1] H-NMR spectrum (C_5D_5N, 400 MHz) of 3*β*,16*β*-dihydroxyurs-12-en-11-one (**1**).

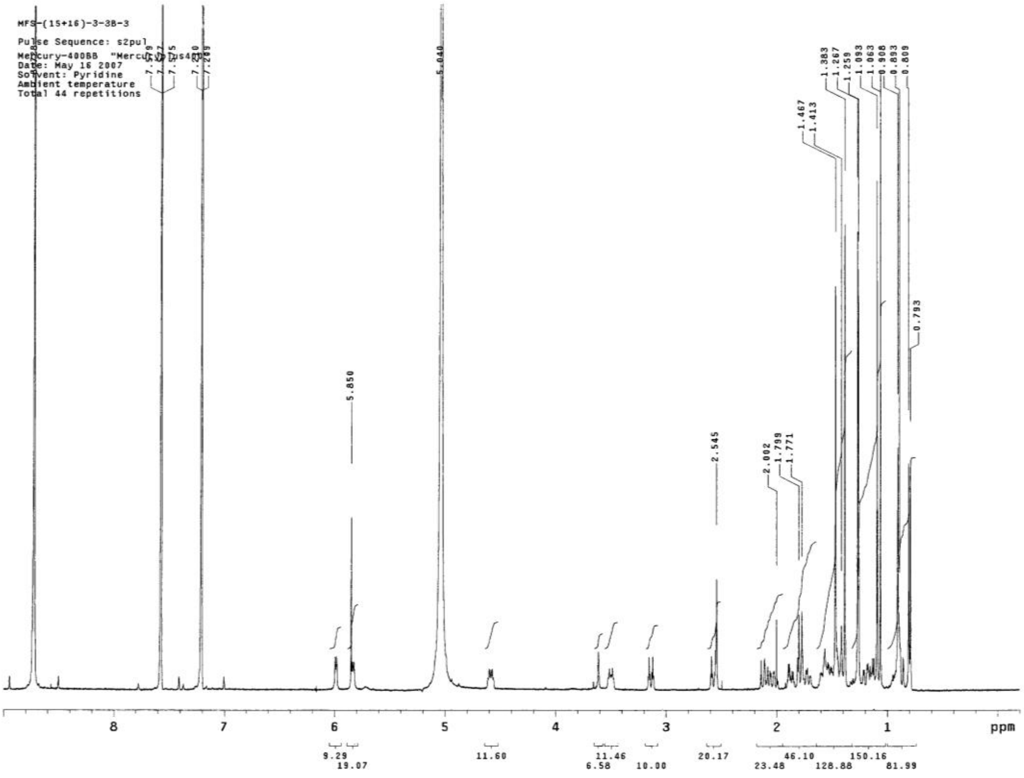

Figure S3. The ^{13}C-NMR spectrum (C_5D_5N, 100 MHz) of 3*β*,16*β*-dihydroxyurs-12-en-11-one (**1**).

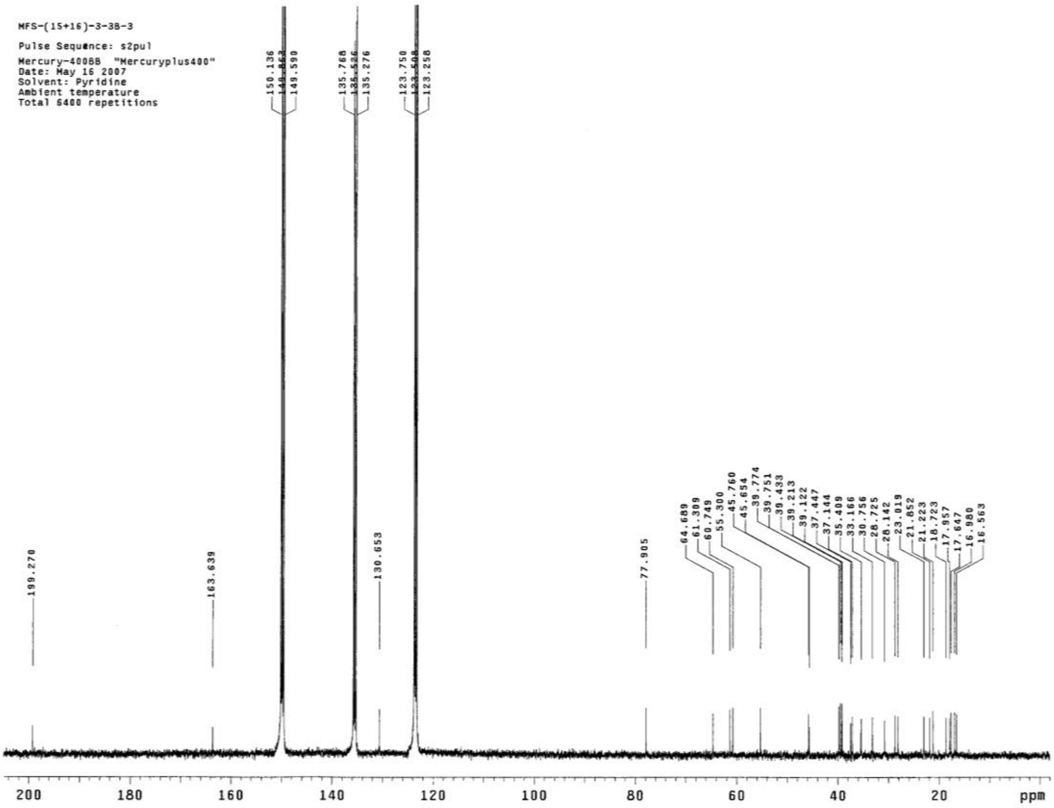

Figure S4. The COSY spectrum of 3*β*,16*β*-dihydroxyurs-12-en-11-one (**1**).

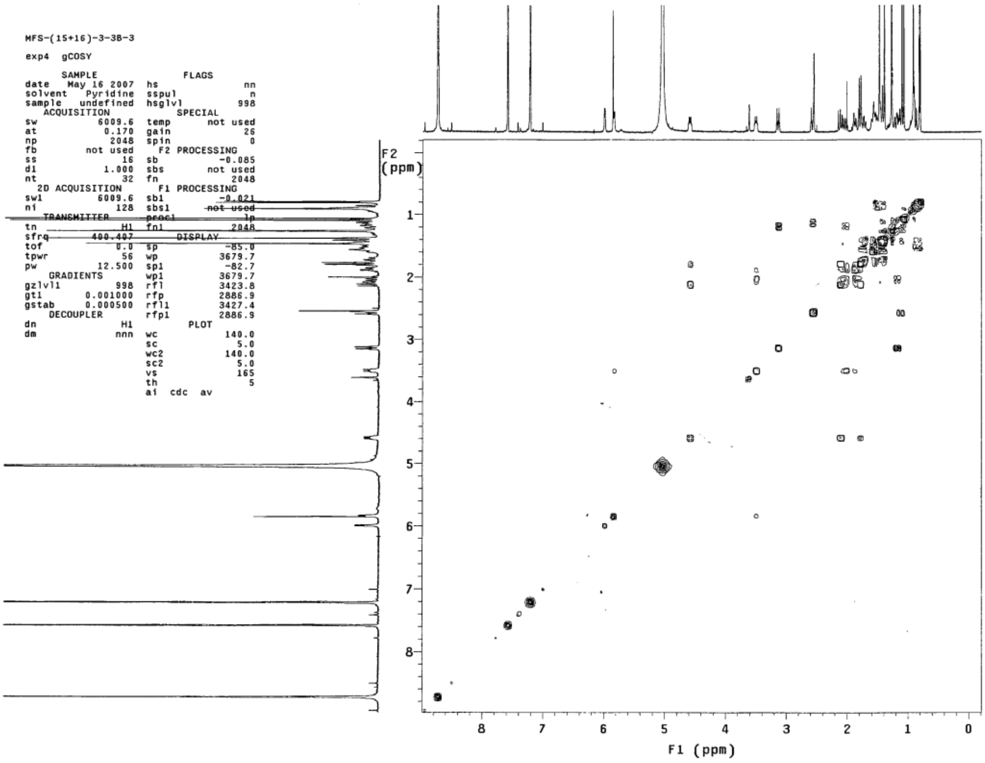

Figure S5. The HMBC spectrum of 3*β*,16*β*-dihydroxyurs-12-en-11-one (**1**).

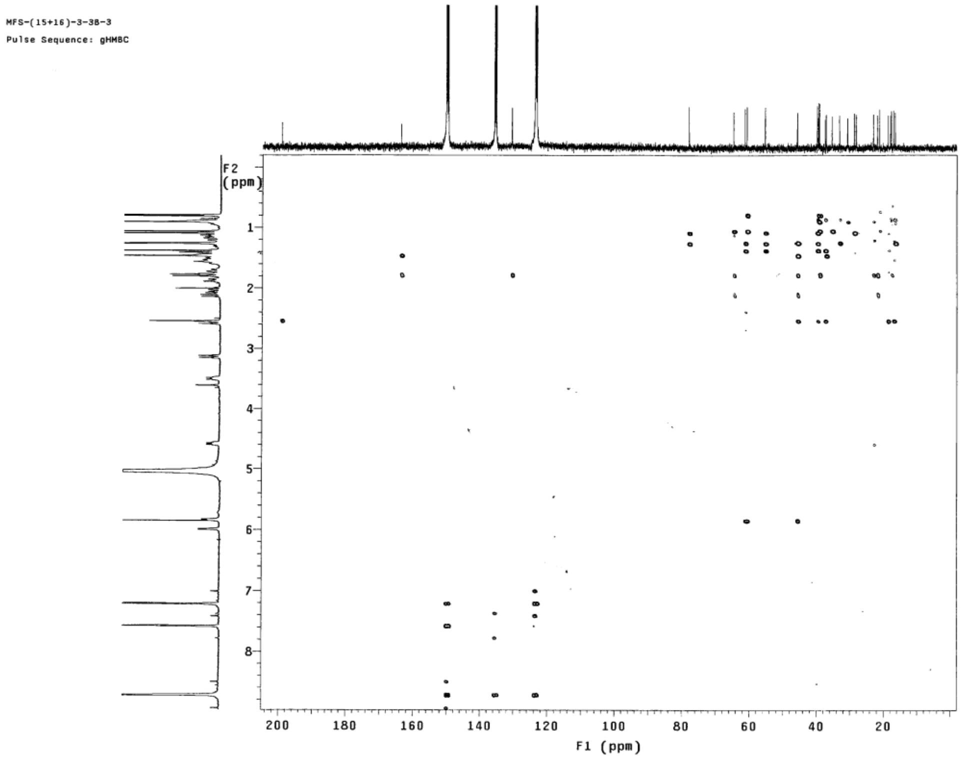

Figure S6. The ^{1}H-NMR spectrum (C_5D_5N, 400 MHz) of 6*β*,12,23-trihydroxy-11 *α*-methoxyurs-12-en-3-one (**2**).

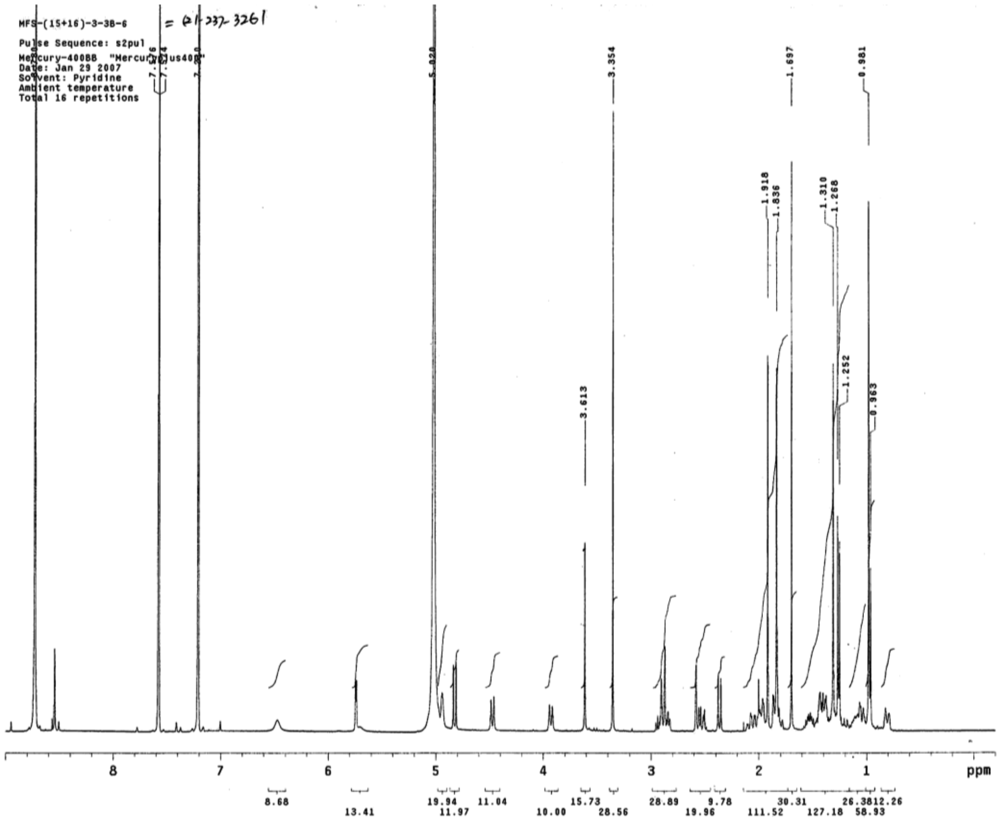

Figure S7. The ^{13}C-NMR spectrum (C_5D_5N, 100 MHz) of 6*β*,12,23-trihydroxy-11 *α*-methoxyurs-12-en-3-one (**2**).

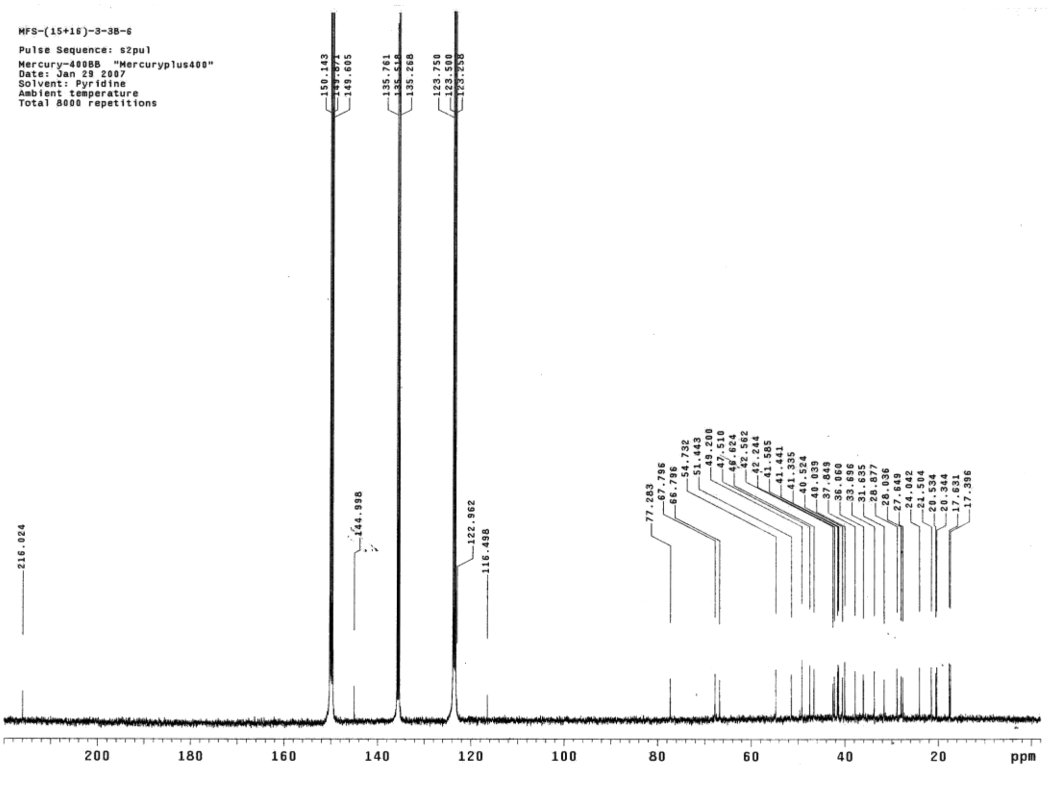

Figure S8. The COSY spectrum of 6*β*,12,23-trihydroxy-11*α*-methoxyurs-12-en-3-one (**2**).

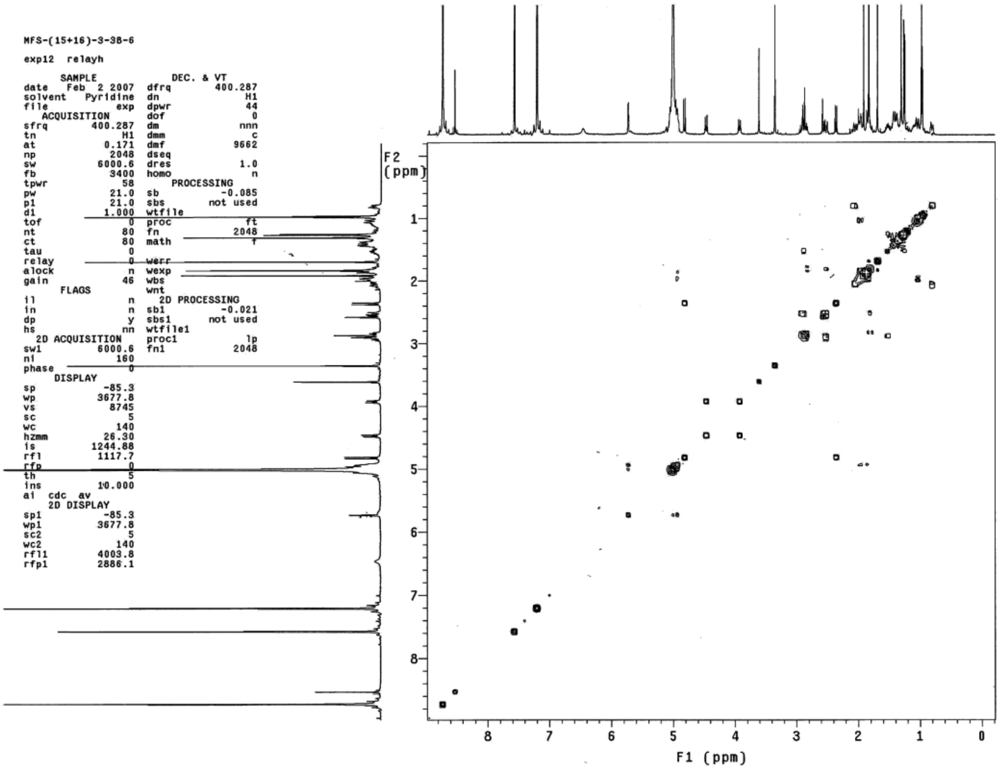

Figure S9. The HMBC spectrum of 6*β*,12,23-trihydroxy-11*α*-methoxyurs-12-en-3-one (**2**).

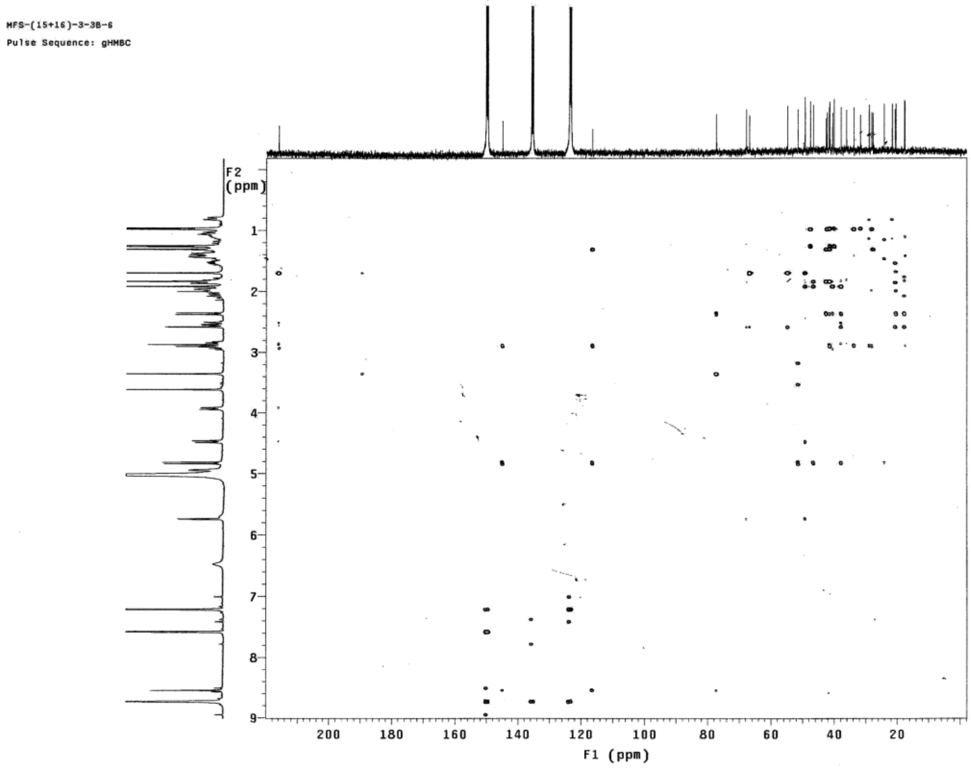

Figure S10. The ^{1}H-NMR spectrum (C_5D_5N, 500 MHz) of 11*α*,12,16*β*-trihydroxyurs-12-en-3-one (**3**).

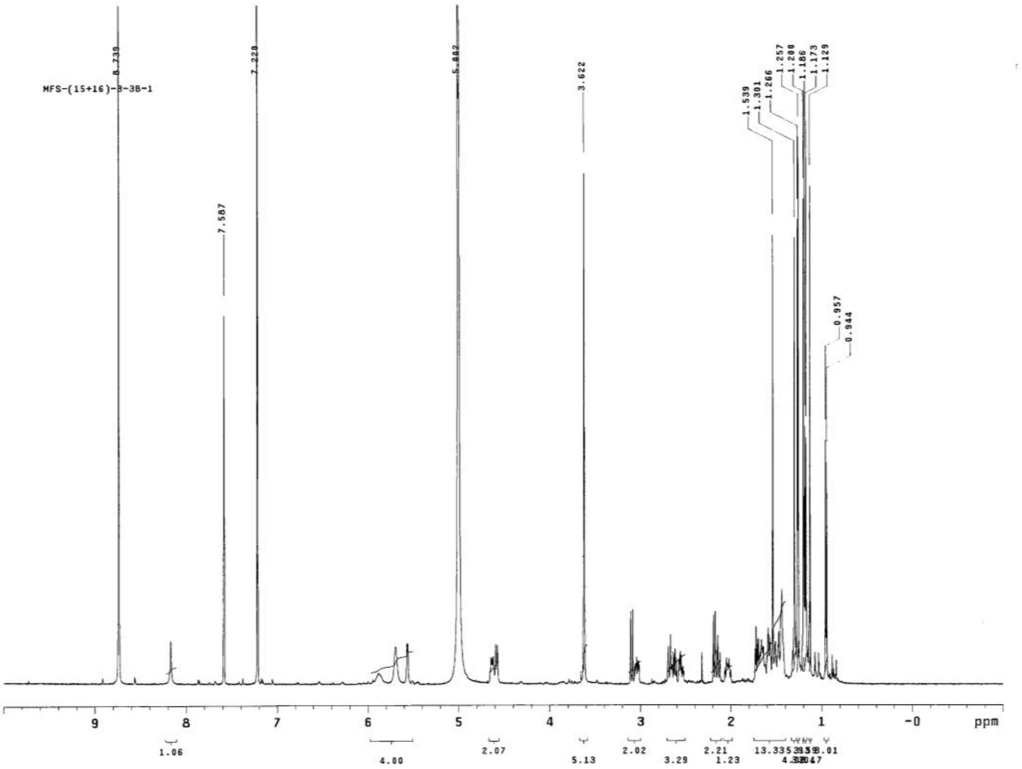

Figure S11. The ^{13}C-NMR spectrum (C_5D_5N, 125 MHz) of 11*α*,12,16*β*-trihydroxyurs-12-en-3-one (**3**).

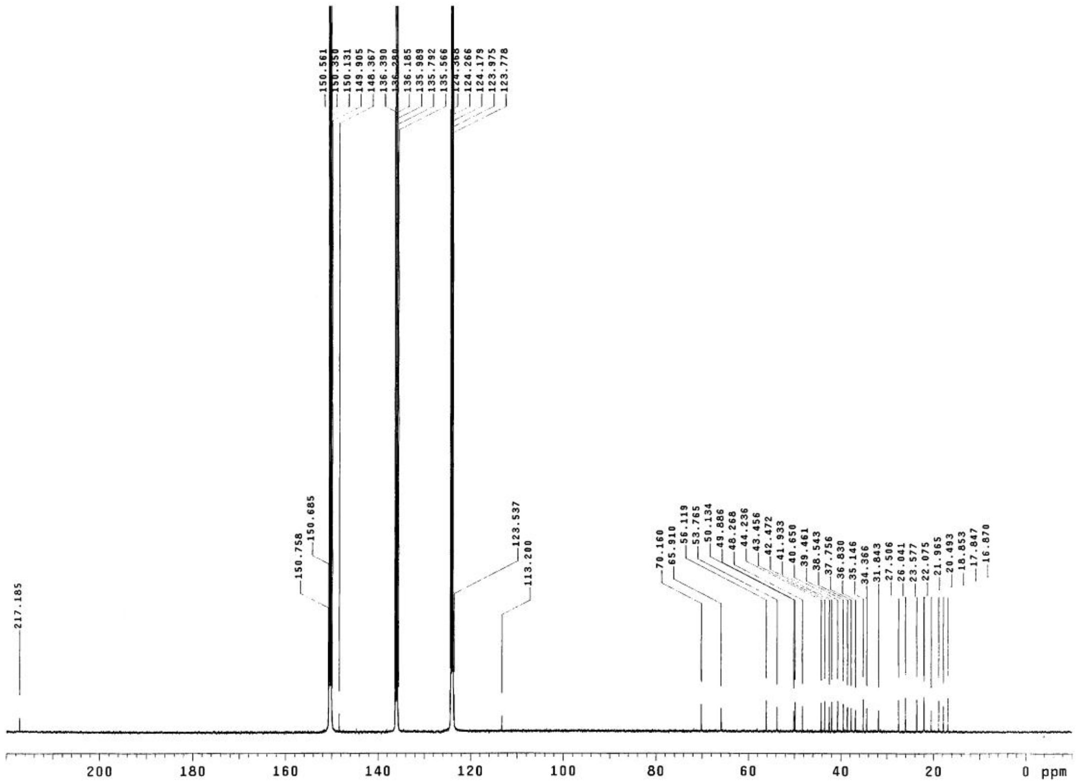

Figure S12. The COSY spectrum of 11α,12,16β-trihydroxyurs-12-en-3-one (**3**).

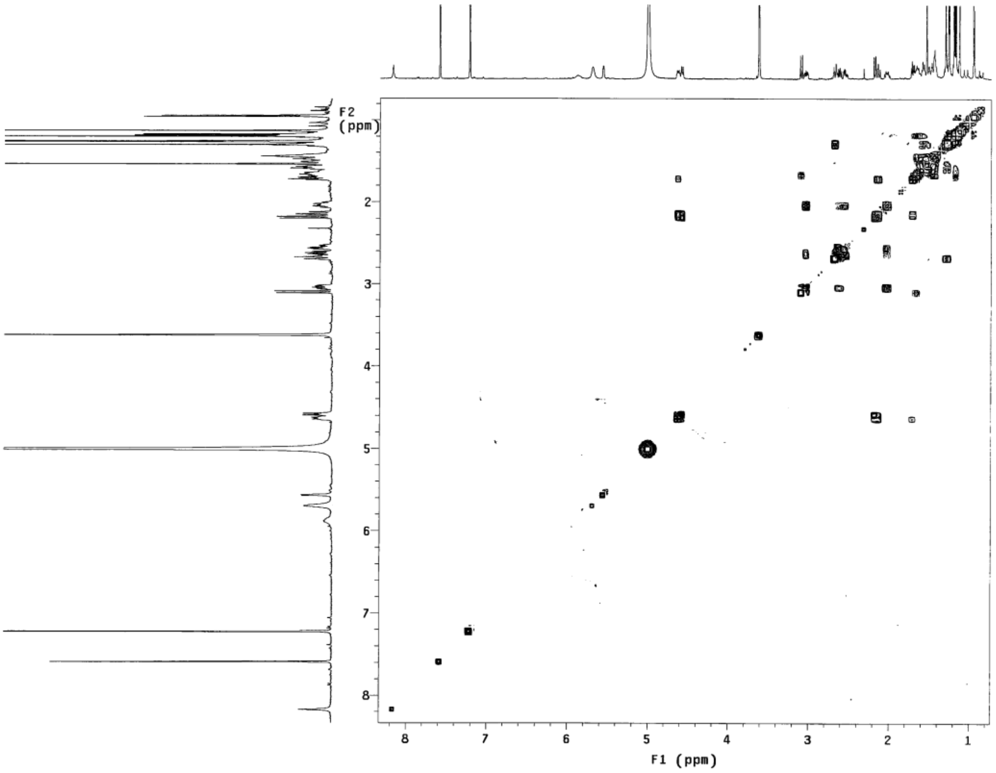

Figure S13. The HMBC spectrum of 11α,12,16β-trihydroxyurs-12-en-3-one (**3**).

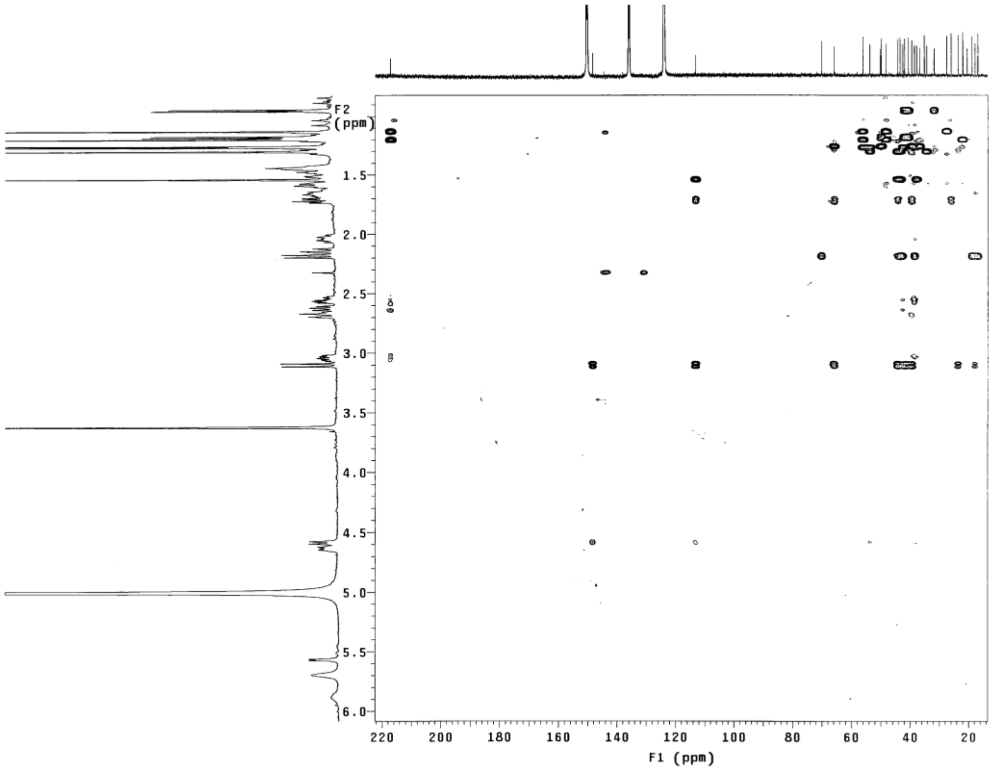

Figure S14. The ^{1}H-NMR spectrum (C_5D_5N, 400 MHz) of 1*α*,3*β*-dihydroxyolean-12-en-11-one (**4**).

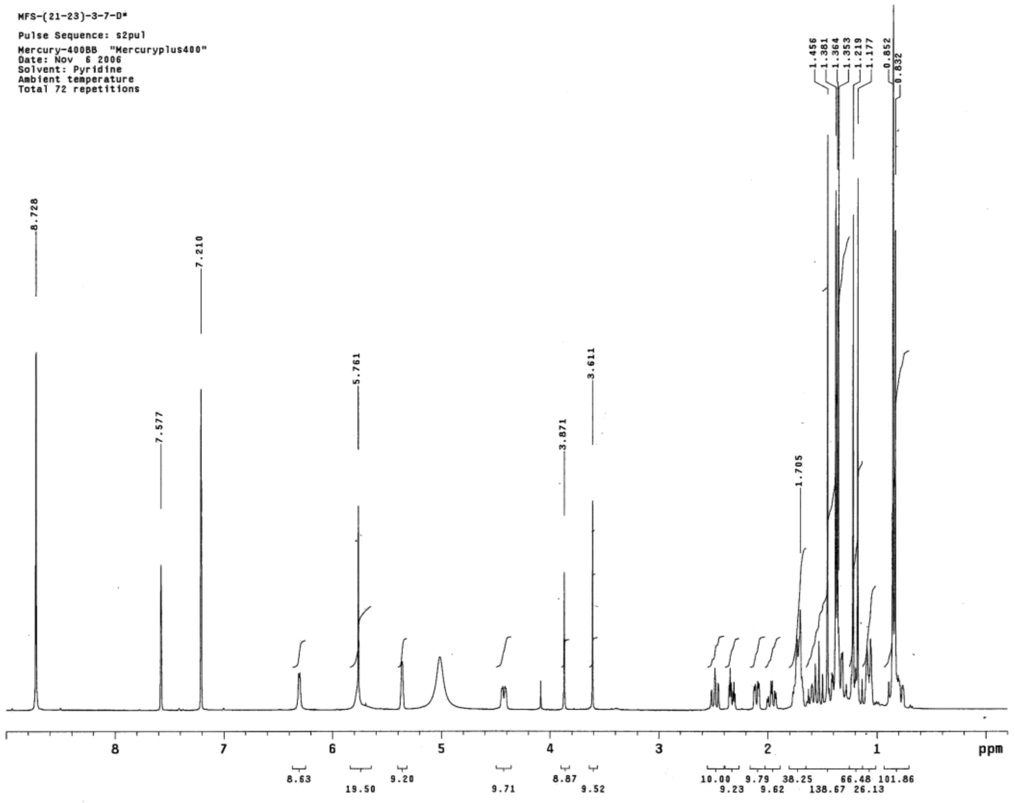

Figure S15. The ^{13}C-NMR spectrum (C_5D_5N, 100 MHz) of 1*α*,3*β*-dihydroxyolean-12-en-11-one (**4**).

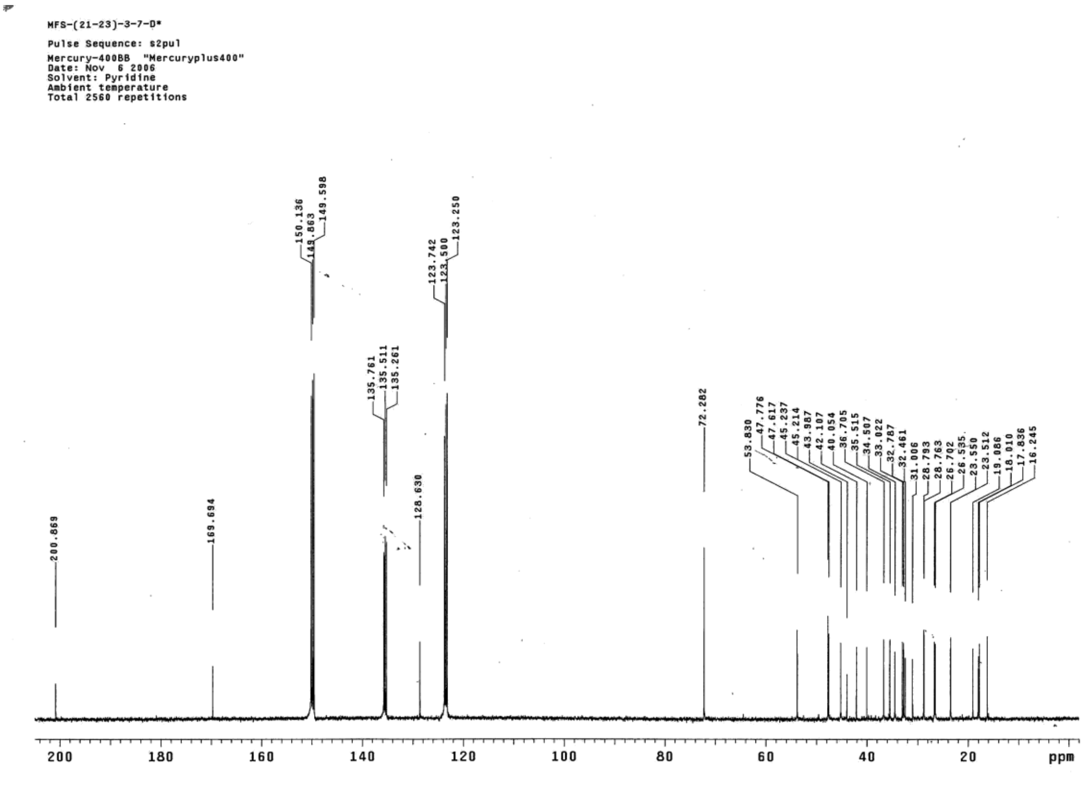

Figure S16. The COSY spectrum of 1α,3β-dihydroxyolean-12-en-11-one (**4**).

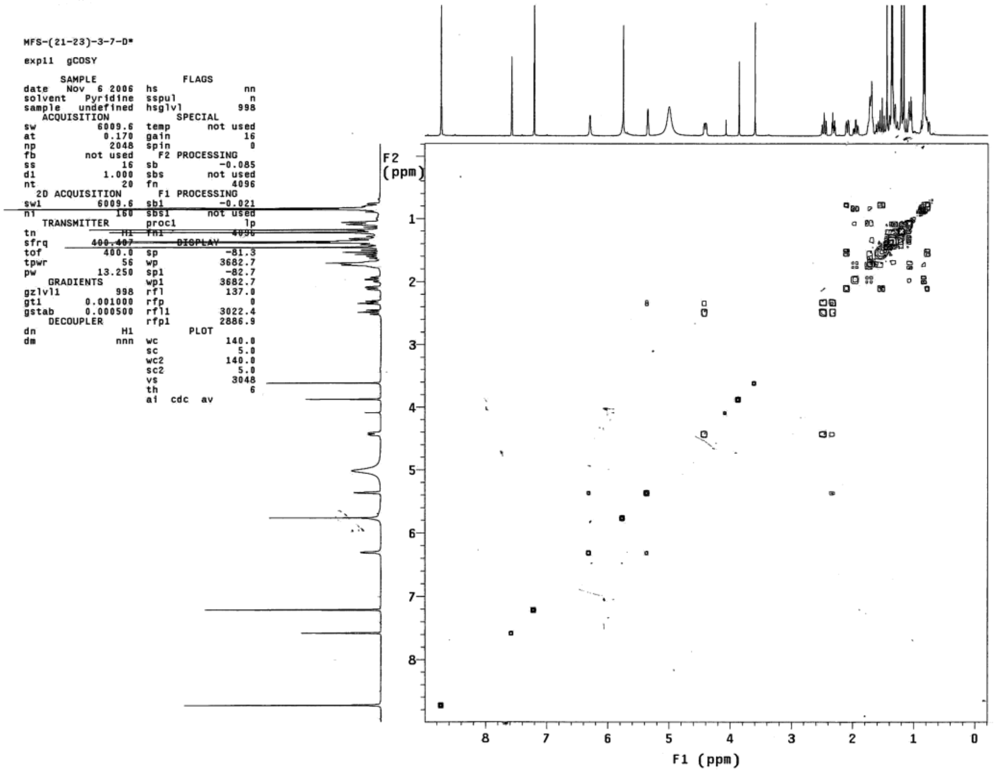

Figure S17. The HMBC spectrum of 1α,3β-dihydroxyolean-12-en-11-one (**4**).

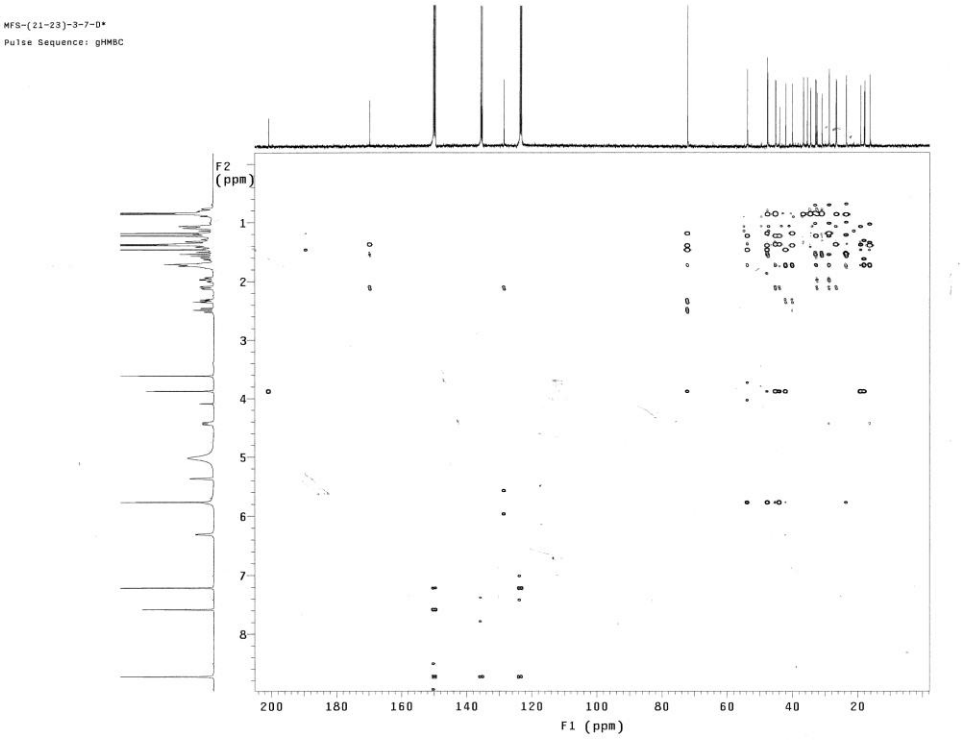

Figure S18. The ^{1}H-NMR spectrum (C_5D_5N, 400 MHz) of 30-hydroxyolean-12-en-3, 11-dione (**5**).

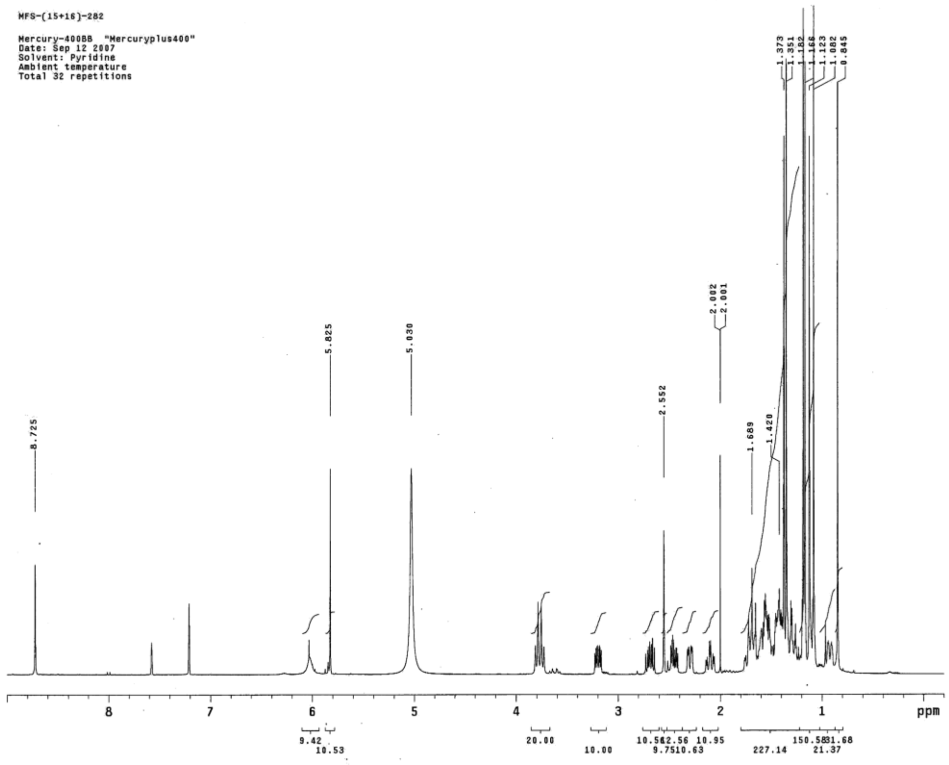

Figure S19. The ^{13}C-NMR spectrum (C_5D_5N, 100 MHz) of 30-hydroxyolean-12-en-3, 11-dione (**5**).

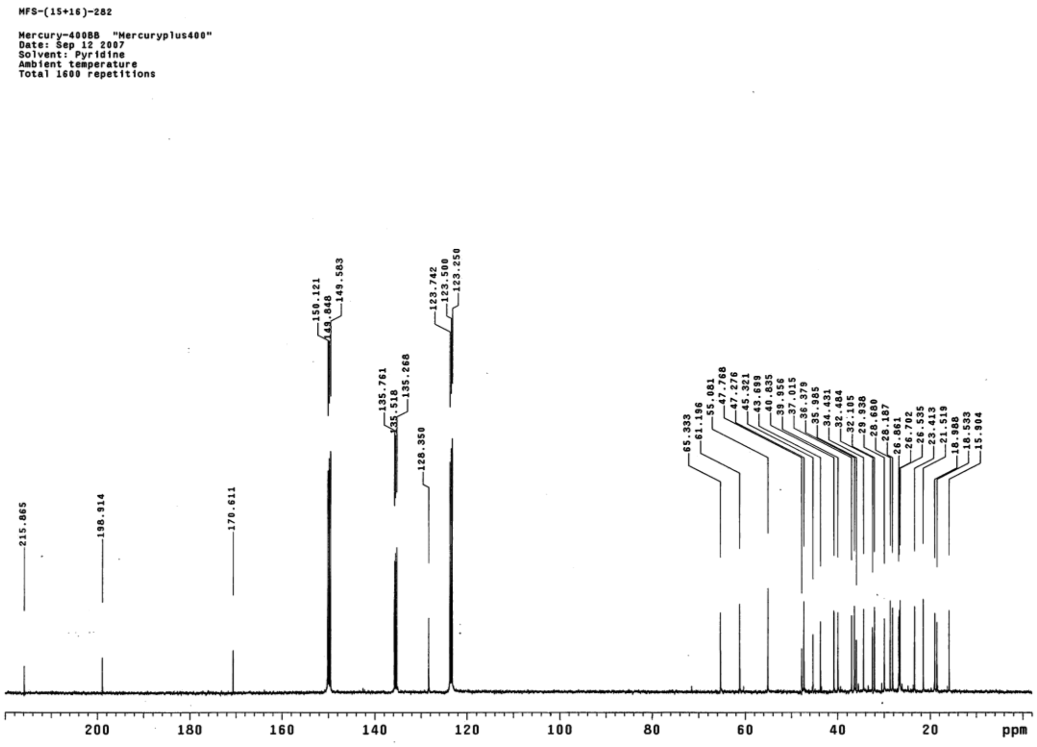

Figure S20. The COSY spectrum of 30-hydroxyolean-12-en-3,11-dione (**5**).

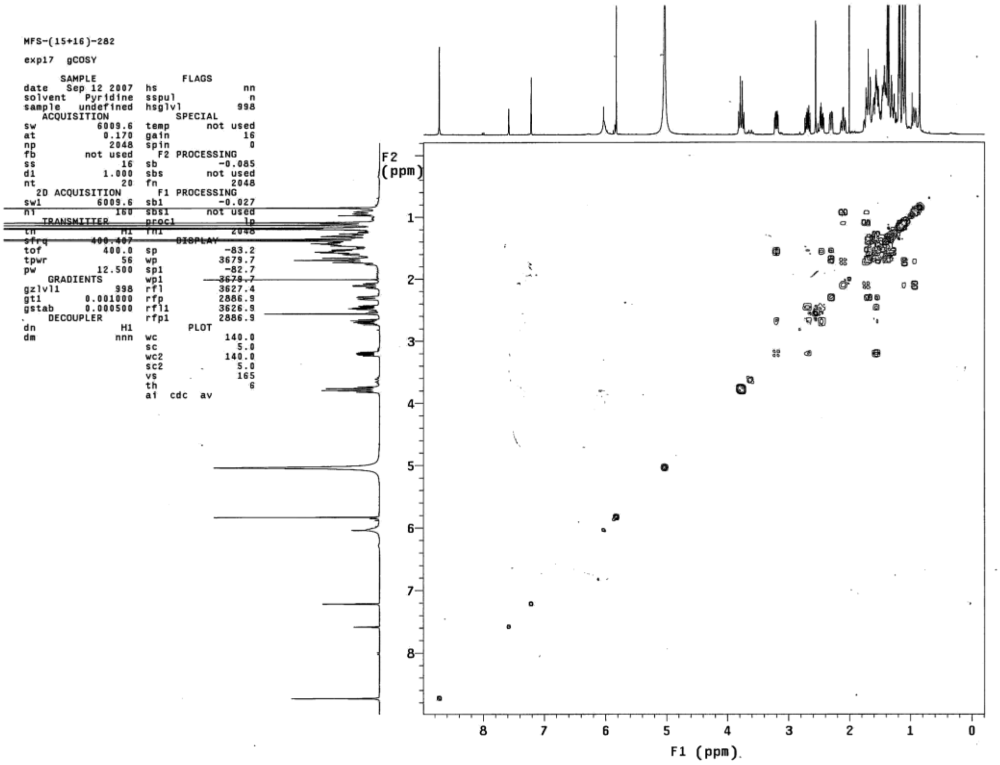

Figure S21. The HMBC spectrum of 30-hydroxyolean-12-en-3,11-dione (**5**).

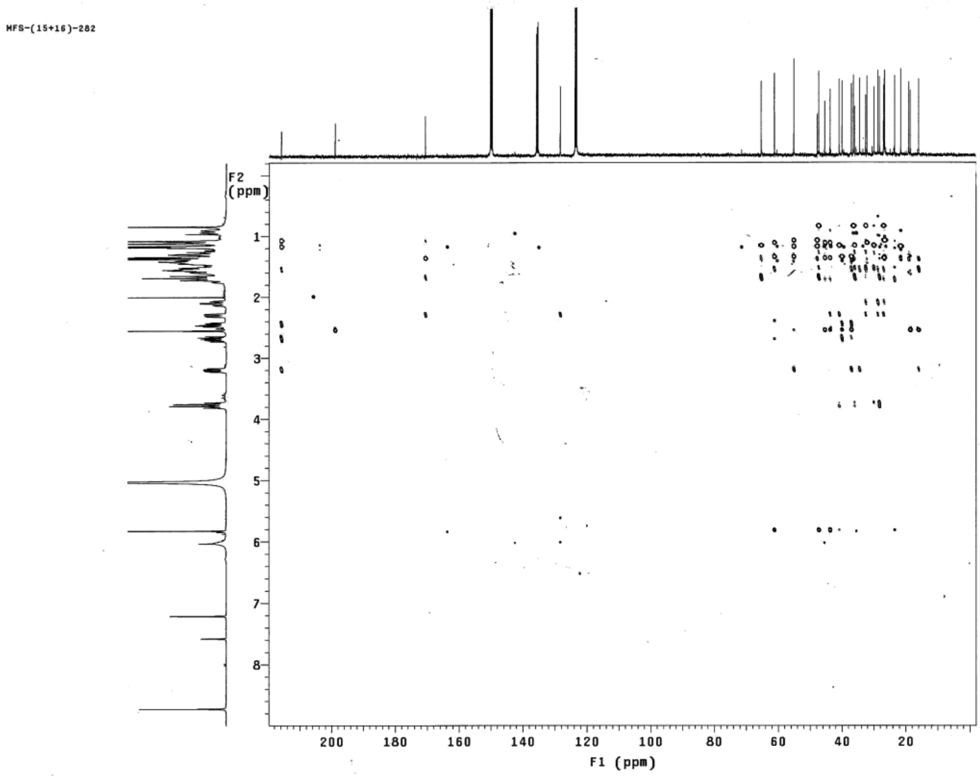

Figure S22. The ^{1}H-NMR spectrum (C_5D_5N, 400 MHz) of 3β,28-dihydroxyolean-18-en-1-one (**6**).

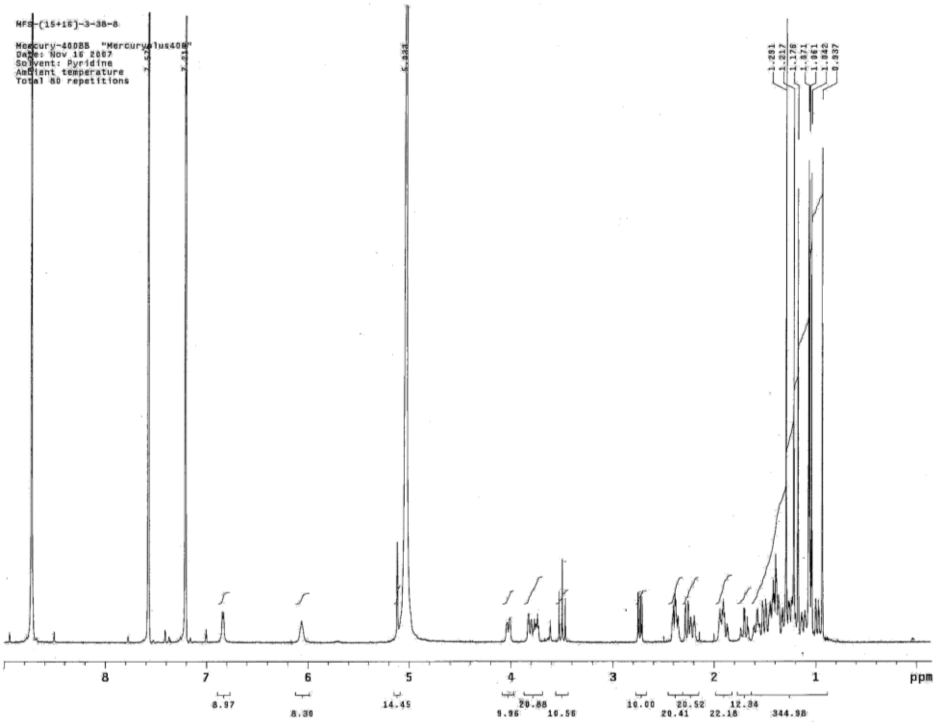

Figure S23. The ^{13}C-NMR spectrum (C_5D_5N, 100 MHz) of 3β,28-dihydroxyolean-18-en-1-one (**6**).

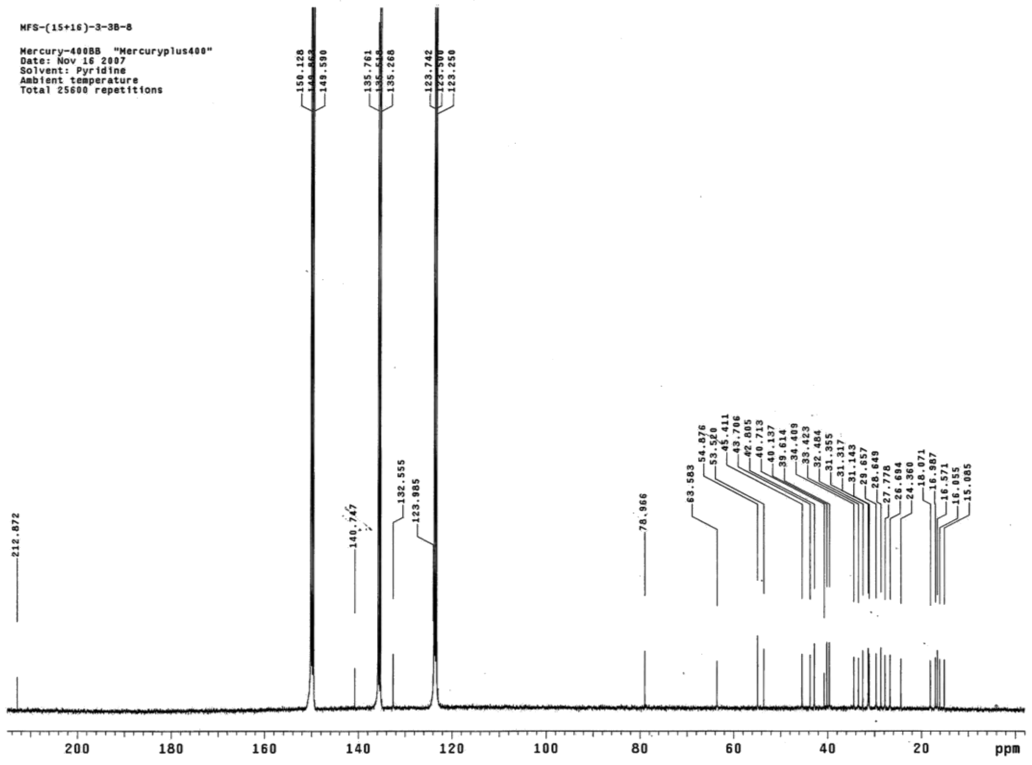

Figure S24. The COSY spectrum of 3β,28-dihydroxyolean-18-en-1-one (**6**).

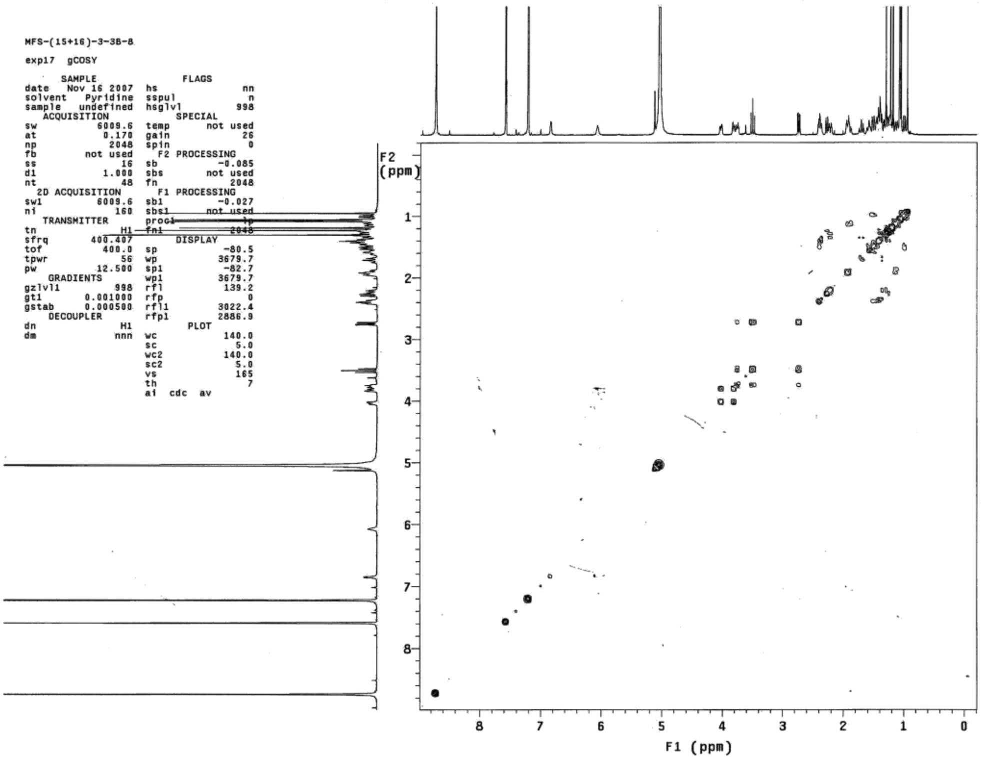

Figure S25. The HMBC spectrum of 3β,28-dihydroxyolean-18-en-1-one (**6**).

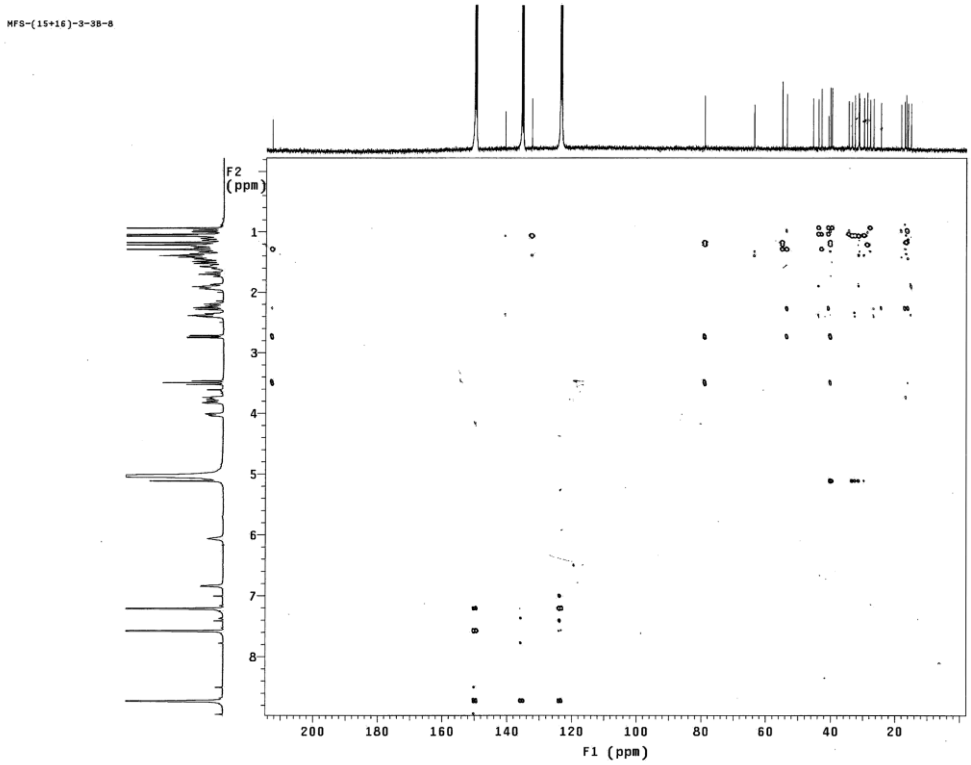

Figure S26. The ^{1}H-NMR spectrum (C_5D_5N, 400 MHz) of 11α,30-dihydroxy-2, 3-*seco*-olean-12-en-2,3-dioic anhydride (**7**).

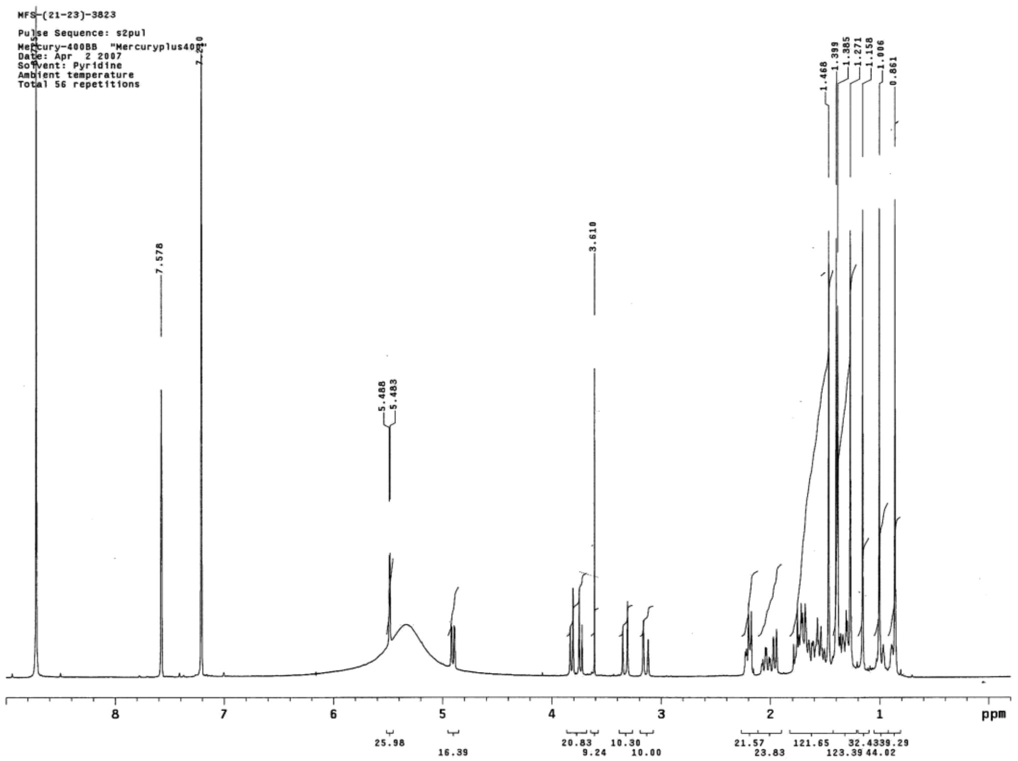

Figure S27. The ^{13}C-NMR spectrum (C_5D_5N, 100 MHz) of 11α,30-dihydroxy-2, 3-*seco*-olean-12-en-2,3-dioic anhydride (**7**).

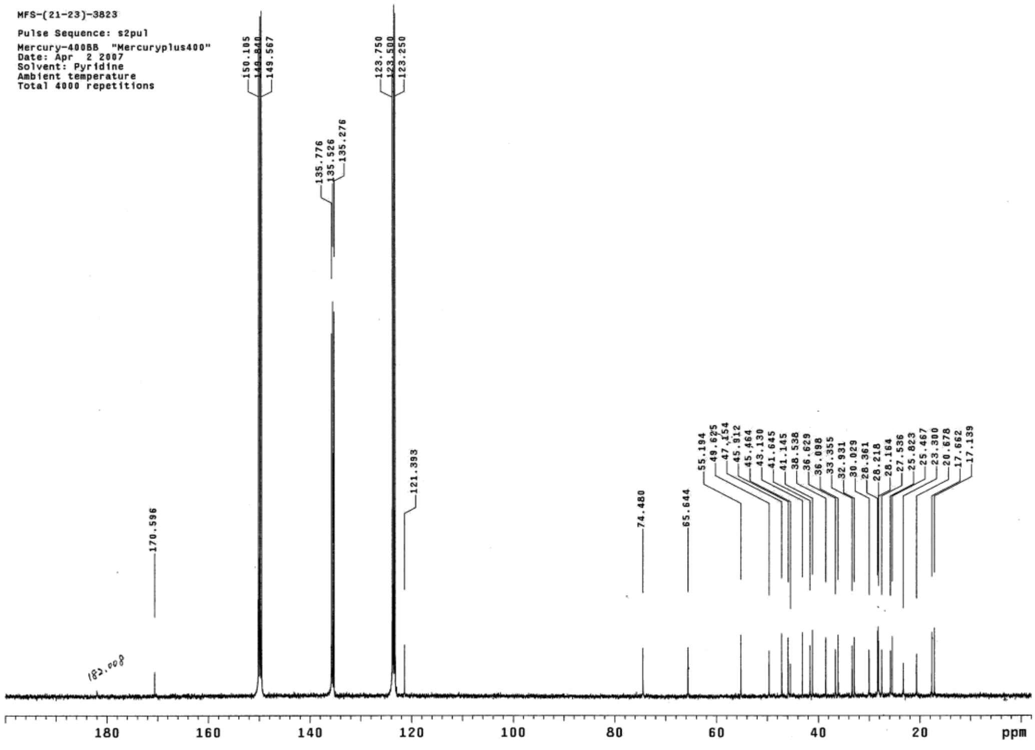

Figure S28. The COSY spectrum of 11α,30-dihydroxy-2,3-*seco*-olean-12-en-2,3-dioic anhydride (**7**).

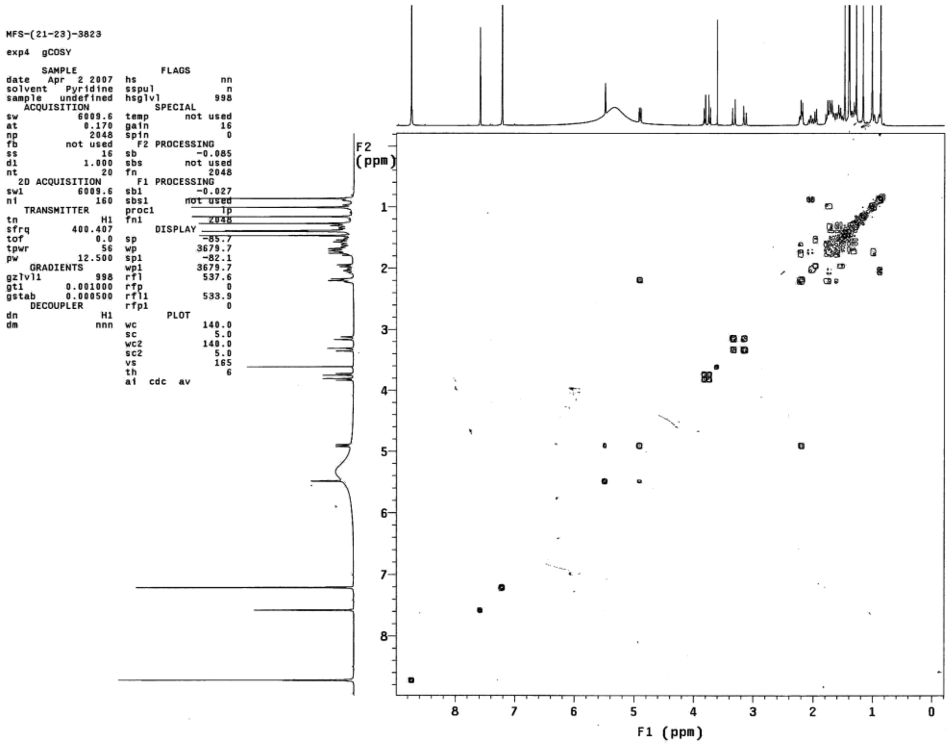

Figure S29. The HMBC spectrum of 11α,30-dihydroxy-2,3-*seco*-olean-12-en-2,3-dioic anhydride (**7**).

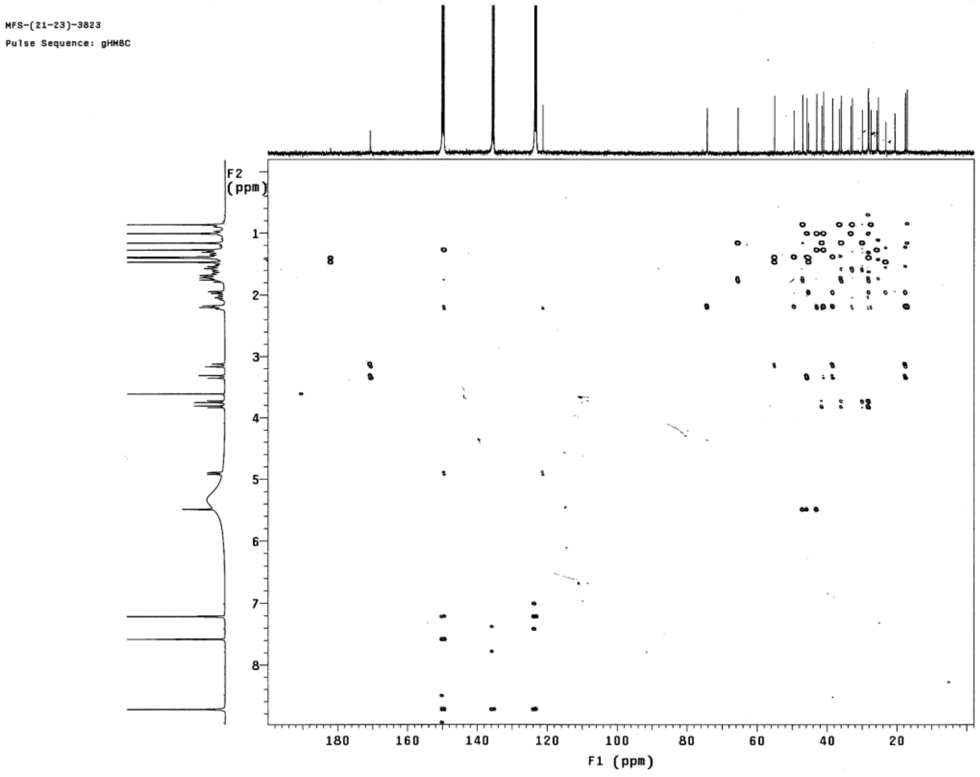

Supplement: Supplementary file 1 [file molecules-19-04608-s001.pdf]
